# Three-dimensional isotropic imaging of live suspension cells enabled by droplet microvortices

**DOI:** 10.1073/pnas.2408567121

**Published:** 2024-10-22

**Authors:** Braulio Cardenas-Benitez, Richard Hurtado, Xuhao Luo, Abraham P. Lee

**Affiliations:** ^a^Department of Biomedical Engineering, University of California, Irvine, CA 92697; ^b^Center for Advanced Design & Manufacturing of Integrated Microfluidics, University of California, Irvine, CA 92697; ^c^Department of Mechanical and Aerospace Engineering, University of California, Irvine, CA 92697

**Keywords:** 3D live-cell imaging, single-cell analysis, microfluidics, nucleus morphology, droplet microvortices

## Abstract

Fast, nondestructive three-dimensional (3D) imaging of live suspension cells remains challenging without substrate treatment or fixation, precluding scalable single-cell morphometry with minimal alterations. While optical sectioning techniques achieve 3D live cell imaging, lateral versus depth resolution differences further complicate analysis. We present a scalable microfluidic method capable of 3D fluorescent isotropic imaging of live, nonadherent cells suspended inside picoliter droplets with high-speed single-cell volumetric readout (800 to 1,200 slices in 5 to 8 s) and near-diffraction limit resolution (~216 nm). The platform features a droplet trap array that leverages flow-induced droplet interfacial shear to generate intradroplet microvortices, which rotate single cells on their axis to enable optical projection tomography (OPT)-based imaging. This allows gentle (~1 mPa shear stress) observation of cells encapsulated inside nontoxic isotonic buffer droplets, facilitating scalable OPT acquisition by simultaneous spinning of hundreds of cells. We demonstrate 3D imaging of live myeloid and lymphoid cells in suspension, including K562 cells, as well as naive and activated T cells—small cells prone to movement in their suspended phenotype. Our fully suspended, orientation-independent cell morphometry, driven by isotropic imaging and spherical harmonic analysis, enabled the study of primary T cells across various immunological activation states. This approach unveiled six distinct nuclear content distributions, contrasting with conventional 2D images that typically portray spheroid and bean-like nuclear shapes associated with lymphocytes. Our arrayed-droplet OPT technology is capable of isotropic, single live-cell 3D imaging, with the potential to perform large-scale morphometry of immune cell effector function states while providing compatibility with microfluidic droplet operations.

## Significance

Nonadherent cells, such as hematopoietic cell types, are cultured and handled in their suspended phenotype as part of standard cell biology protocols. In that state, sample drift precludes systematic three-dimensional (3D) imaging and screening of suspended cell populations, unless cells are fixed, attached to flat surfaces, or imaged with high temporal resolution. Our microfluidic platform, facilitated by the encapsulation of single cells in droplets, controllably rotates them in an arrayed format to individually inspect cells over 360° while free-floating. This enables fast acquisition (~5 to 8 s per cell) of live 3D single-cell fluorescence data, while allowing cell shape analysis independent of their suspended orientation, thereby providing a framework for systematically studying the morphology of populations of suspended cells.

Advances in high-content single-cell microscopy, typically based on optical sectioning technologies (1), have transformed the biomedical field. Notable examples include elucidating the structure of the immunological synapse in T cells (2) and visualizing the 3D spatial configuration of intracellular components, such as the nucleus (3). While confocal laser scanning microscopy (CLSM) dominates the 3D live cell imaging field, the method suffers from poor temporal resolution, volumetric bleaching, and significant depth-dependent signal degradation even across the volume of a single cell (4). Structured illumination microscopy (SIM) and lattice light-sheet microscopy (LLSM) alternatively offer higher spatiotemporal resolution, albeit at increased instrumentation complexity and cost. Moreover, overarching limitations include the low throughput of imaging and the inability to image suspended, nonadherent single cells without modifying the surface of the imaging substrate (5). Suspended cell populations, like lymphocytes of the immune system, therefore, present a unique 3D imaging challenge due to their tendency to drift without substrate attachment. To minimize motion artifacts for high-resolution live cell topographic and intracellular morphometric measurements (6), isotropic and depth-independent signal acquisition is therefore critical.

Flow cytometry techniques using light-sheet illumination (5) and tomographic imaging (7) have advanced 3D single-cell analysis at the population level but entail costly optical instrumentation and face resolution limitations [~0.5 to 1 µm, with proposed slower flow rates to approach the optical limit (5)]. Recently, light-field flow cytometry has also been explored as a high-throughput 3D imaging alternative but faces similar resolution limitations (400 to 600 nm) (8). While these flow cytometry–based techniques represent the state of the art for high-throughput 3D volumetric cell screening, flow conditions do not provide control over single cells, preclude time-dependent analysis, and the shared fluidic environment increases the chance of diffusive cross talk between cells, while also impeding cell compartmentalization with soluble factors of choice.

Volumetric imaging methods based on optical projection tomography (OPT) have been proposed to image single cells in their suspended phenotype (9) and with spatial isotropic resolution at the diffraction limit (6). In OPT, a series of 2D projections of a sample is acquired from different angles, similar to the process used in computed tomography (6). These projections are then backprojected into 3D space from the angles they were taken, effectively reversing the process of generating the 2D projections from the object. A reconstruction algorithm, often incorporating Fourier domain filters like the ramp filter, is then used to combine and refine all the backprojected images, thereby reconstructing the internal 3D structure of the sample. In epifluorescence Live Cell Computed Tomography (LCCT), single cells are rotated to acquire 2D pseudoprojections, which has been used to produce 3D isotropic images of mitochondrial and nuclear dynamics in suspension cell lines (6). Highly inclined and laminated fluorescence computed tomography (HILO-fCT), another variation of LCCT, offers reduced photobleaching and improved image contrast by using optical sheets instead of epifluorescence excitation (10). While both LCCT and HILO-fCT are capable of suspended cell 3D imaging, they operate at low throughputs and require dielectrophoretic single-cell handling via micropatterned electrodes, which optimally operates at low conductivity buffers (~10 to 100 mS m^−1^) to minimize cell membrane damage via Joule heating (11, 12). In contrast, microfluidic cell rotation methods provide less restrictive liquid media conditions and higher throughput. Hydrodynamically induced cell rotation has been used for quantitative phase imaging (13, 14), and acoustically driven bubble arrays have demonstrated large-scale single-cell rotation compatible with tomographic reconstruction (15, 16). Furthermore, methods demonstrating the use of droplet microfluidics platforms for 3D spatial tissue patterning with potential for multiangle observation and quantitative image analysis have been explored, albeit with limited 3D imaging capabilities stemming from the use of large working distance objectives (17). Therefore, while numerous microdevice-based cell rotation techniques exist (18), there is still a need for methods that offer 1) parallel single-cell rotation, enabling 3D isotropic optical analysis of cell populations at the diffraction limit; 2) compatibility with label-specific fluorescence imaging; and 3) an individualized microfluidic environment for live, unfixed cells, especially those prone to movement, such as suspended cells (e.g., immune cells).

**Fig. 1.**
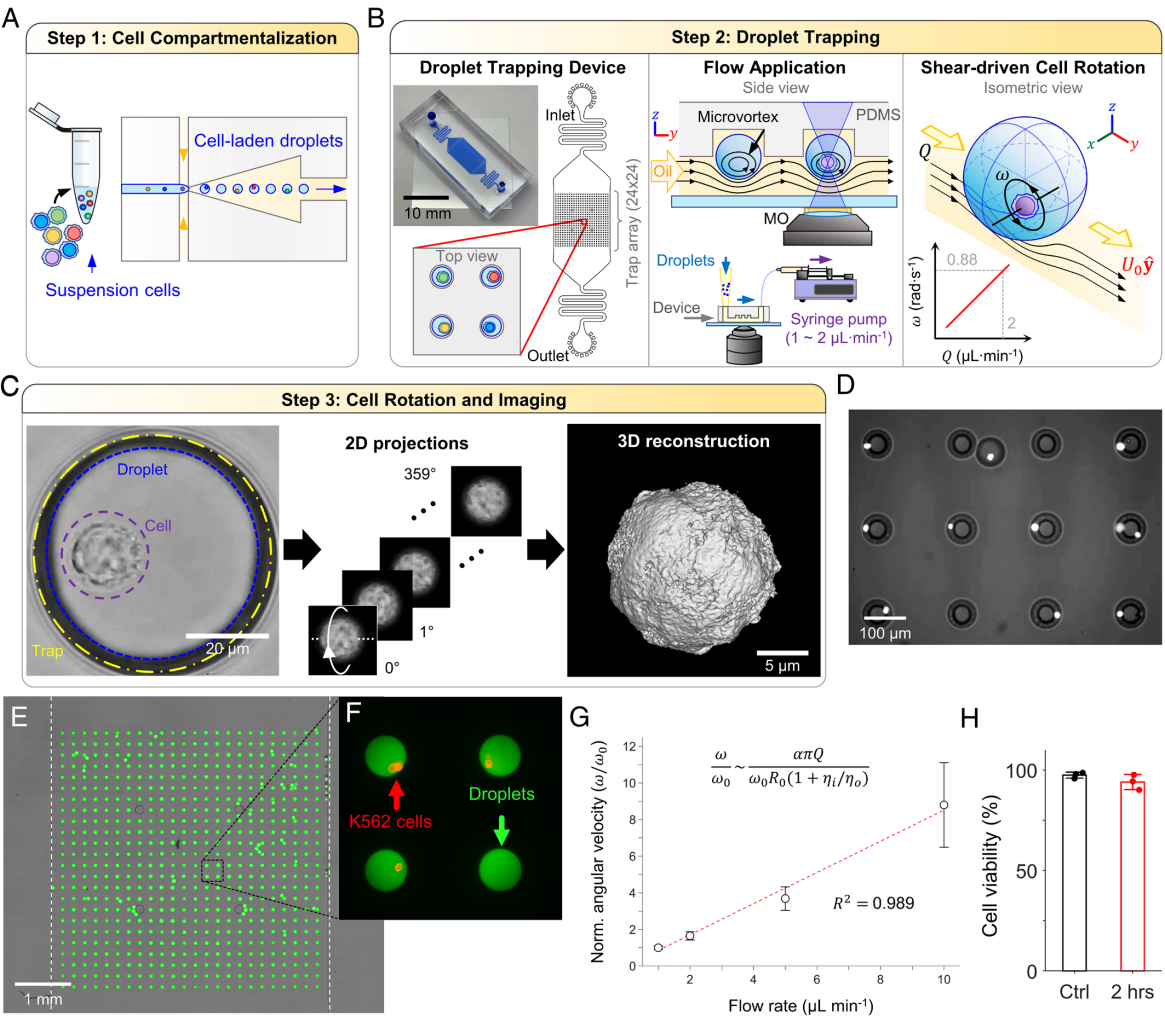
ADOPT Workflow and operation. (*A*) A structurally heterogeneous population of cells is first compartmentalized into water-in-oil droplets. (*B*) Cell-laden droplets are then loaded into a PDMS microfluidic trap array (24×24). Each trap consists of an inverted, circular microwell that can trap exactly one cell-laden droplet. Continuous oil perfusion transfers shear stress to the inside of droplets, generating recirculation microvortices that drive cell self-rotation. The application of flow rate (1 to 2 µL min^−1^) leads to linear increases in flow velocity (80 to 160 µm s^−1^) and shear forces at the droplet surface (2.1 to 4.2 mPa), which in turn linearly drive cell angular velocity (0.44 to 0.88 rad s^−1^). (*C*) Time-lapse frame acquisition of the rotating cells allows their full 360° observation, which can be used to approximate their 3D structure through point spread function (PSF)-informed OPT reconstruction algorithms. (*D*) Micrograph of multiple droplets containing rotating K562 cells stained with Hoechst 33342 dye. (*E*) Microfluidic trap array image obtained via image stitching, showcasing 100% loaded traps with 95% single droplet occupancy. Fluorescein dextran was used to enhance droplet visualization. (*F*) Blow-up of (*E*), displaying four droplets with stained suspension cells (K562, anti-CD45-PE). (*G*) Normalized angular velocity of encapsulated cells as a function of external oil flow rate. (*H*) K562 cell viability inside droplets when subjected to 2 h of continuous oil flow rate (1 µL min^−1^).

Here, we present a passive microfluidic droplet trap array that leverages oil flow-induced interfacial shear to generate intradroplet microvortices (Fig. 1 *A*–*C*). These microvortices, in turn, mobilize encapsulated cells inside the water-in-oil droplets at an angular velocity determined by the exterior phase applied flow rate (Fig. 1*G*). This strategy allows observation of cells encapsulated inside picoliter droplets of conventional, nontoxic isotonic buffer and facilitates scalable, arrayed OPT acquisition by the simultaneous spinning of cells (Fig. 1 *D* and *E*). Our arrayed-droplet optical projection tomography (ADOPT) imaging modality enabled the isotropic 3D imaging of live myeloid and lymphoid cells in suspension and at the optical diffraction limit (x, y, z resolutions ~216 nm with λ = 461 nm, NA = 1.3). The method allowed single-cell morphometric analysis using a spherical harmonic (SPHARM) based approach, which was applied to study K562 cells, as well as naive and activated healthy-donor derived T cells—immune cells prone to motion in their suspended state. This facilitated orientation-independent nuclear content morphometry, which identified six distinct nuclear shape categories across immunological activation states over the course of 7 d. Furthermore, ADOPT demonstrated intracellular and fluorescence surface imaging of cells using a simple, camera-based epifluorescence microscopy setup. Owing to this camera-based, single-cell continuous signal recording strategy, we were able to perform pixel-to-pixel image restoration through temporal filtering of photon influx events damaged by noise. This approach significantly improved noise characteristics of the standard complementary metal-oxide semiconductor (CMOS) detector here used, especially at low signal-to-noise (SNR) ratios. Collectively, our findings present a droplet microfluidics framework that enables high-resolution, 3D isotropic analysis of cells in suspension, opening avenues for population-based investigations into their dynamic intracellular and topographic features. Furthermore, this is a demonstration of controlled cell orbiting in stationary droplets that has potential for studying cell biomechanics and cell–cell communication.

## Results

### ADOPT.

**Fig. 2.**
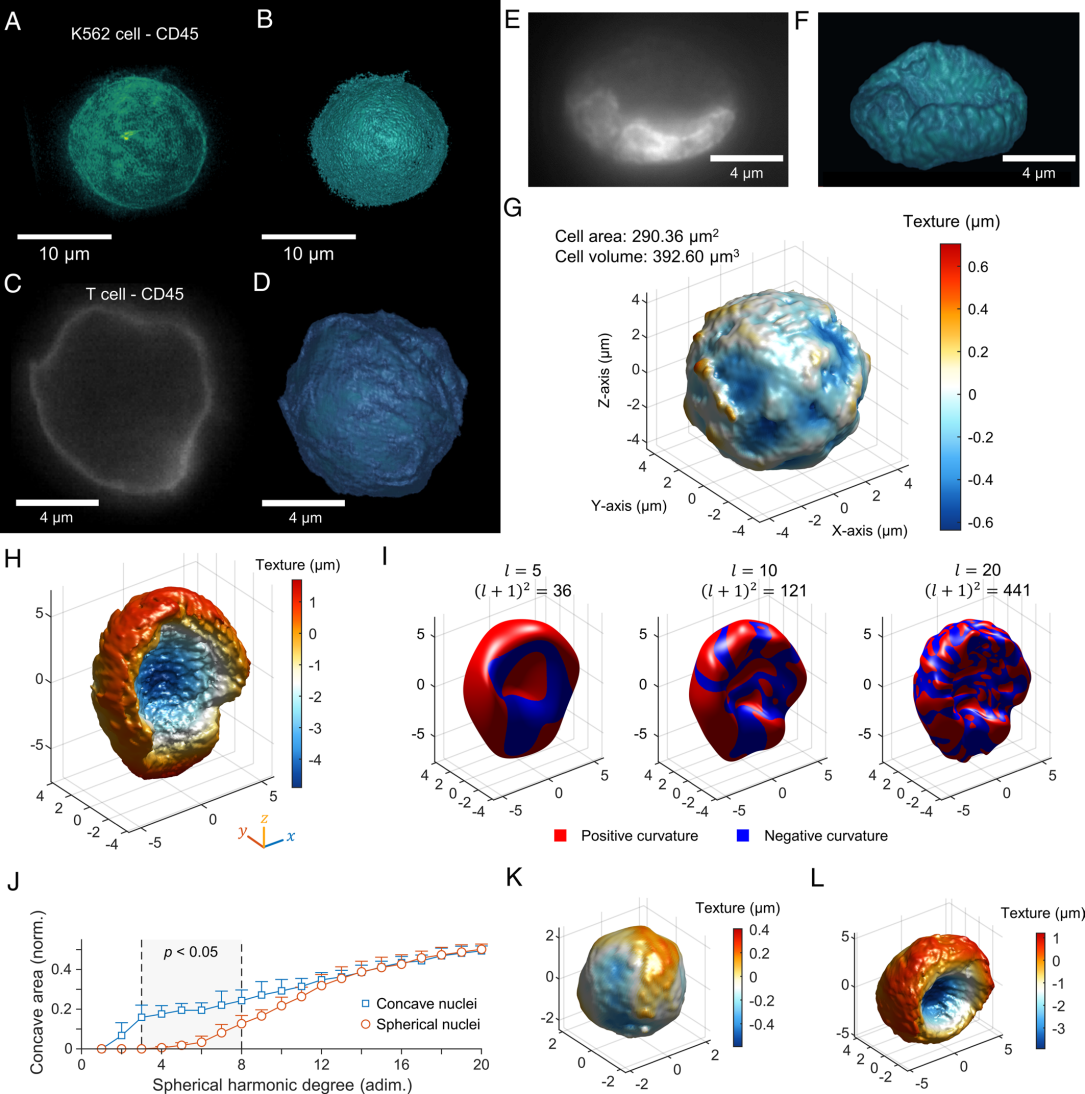
ADOPT enables suspended single-cell 3D isotropic imaging and organelle morphometry. (*A*) Isotropically resolved suspension cells (K562 cell line) stained with anti-CD45 – Alexa Fluor 488, visualized via its MIP. (*B*) Isosurface model of 3D fluorescence data in (*A*). (*C*) Two-dimensional slice of an activated T cell (CD25+), marked with anti-CD45-Alexa Fluor 488 taken during acquisition of the required 360° projections for 3D reconstruction via OPT. (*D*) Isosurface of the CD45 fluorescence level in (*C*). (*E*) Two-dimensional image of the stained nucleus (Hoechst 33342 dye) of a K562 cell. (*F*) Three-dimensional reconstruction of the nuclear content distribution of the nucleus in (*E*). (*G*) Isosurface of the CD45 marker 3D distribution in a K562 cell, detailing quantitative data of its texture with a heatmap. Texture map was defined as the distance between a surface patch and the average radius of the complete isosurface. (*H*) Texture map of the K562 cell nucleus in (*E* and *F*), displaying an enlarged nuclear groove structure. (*I*) Shape approximation of surface model in (*H*) via SPHARMs expansion of increasing degree, l. The total number of harmonic coefficients is given by ${\left(l+1\right)}^{2}$, which accounts for azimuthal components in the expansion. The sign of the Gaussian curvature of each l-degree surface model was computed and displayed on the surface to evince regions of negative and positive curvature. Predominantly concave sections of the shapes were defined to be the portions with negative curvature. (*J*) Plot of the fraction of the nucleus surface area that is concave (negative Gaussian curvature) for a given SPHARM degree expansion, which evinces quantifiable morphological differences between spherical and concave-shaped nuclei. Texture maps of (*K*) spherical and (*L*) concave K562 nuclei.

We present a microfluidic droplet trap array that uses oil flow-induced shear to generate microvortices within droplets, allowing continuous rotation of encapsulated cells for 3D isotropic fluorescence imaging at the optical diffraction limit. This system facilitates a scalable, ADOPT readout, with three key steps: 1) compartmentalization of cells into water-in-oil picoliter droplets using a previously reported microdevice (19) (Fig. 1*A*); 2) passive droplet trapping and hydrodynamic intradroplet microvortex generation (Fig. 1*B*); and 3) large-scale single-cell 360° visualization for OPT 3D reconstruction (Fig. 1*C*).

The droplet array trap device comprises a two-layer flow-through PDMS channel, with the first layer at 40 µm height and the second layer on top at 30 µm height (refer to Fig. 1*B* and *SI Appendix*, Fig. S1 for device side-view). The upper layer features a 24×24 inverted circular microwell array designed for one droplet per trap. Water-in-oil, cell-laden droplets are directed into hydrodynamic traps by perfusing oil at a consistent flow rate. Droplets, with a selected diameter of 65 to 71 µm, appear slightly compressed in the depth direction upon loading into the device. As droplets approach a circular microwell, they are captured through a combination of buoyancy (HFE7500 oil density of 1,614 kg m^−3^) and surface energy minimization (20). In the microfluidic trap array (depicted in Fig. 1 *E* and *F*), a 100% droplet occupancy efficiency with a 95% single droplet trapping rate is achieved. The droplet generation device required as little as 80 to 100 µL of cell suspension to generate cell-laden droplets (*SI Appendix*, Fig. S1). Moreover, the trap array operation and microvortex-driven 3D reconstructions were accomplished with only 10 µL of water-in-oil, cell-laden droplets.

To characterize single-cell rotation dynamics, droplets were loaded with K562 suspension cells (Fig. 1*F*). We determined that external oil flow rate can be used to increase the velocity magnitude of microvortices inside droplets, which in turn modulates the rotation frequency (or equivalently, angular velocity). As seen in Movies S1 and S2, increasing oil flow rate from 2 to 10 µL min^−1^ significantly increased angular velocity. Furthermore, Fig. 1*G* depicts how a 10-fold increase in flow rate (1 to 10 µL min^−1^) results in a roughly ~9-fold angular velocity increment. To explain this approximately linear relationship (*R*^2^ = 0.989), we derived the recirculation timescale (τ) of an infinitesimally small particle following the closed-loop vortex streamlines of an intradroplet Hadamard-Rybczynski flow (21), and applied it to estimate the angular velocity (*SI Appendix*, Fig. S2). This approximation stems from the observation that trapped droplets undergo similar recirculation velocity fields but neglects the intricate effects of trap geometry on flow. We found τ is given by $\tau ∼2R_{0}/U_{0}\left(1+\eta_{i}/\eta_{o}\right)$, where $R_{0}$ is the droplet radius, $U_{0}$ the bulk flow velocity and $\eta_{i}/\eta_{o}$ the ratio of inner and outer phase viscosities (*SI Appendix*). If cell rotation period is T∼τ, and $U_{0}$ is proportional to the applied flow rate $\left(U_{0}∼\alpha Q\right)$, ω becomes

[1] $$\omega \left(Q\right)∼\frac{\alpha \pi Q}{R_{0}(1+\eta_{i}/\eta_{o})}.$$

We further tested the effects of hydrodynamically induced cell rotation on cell viability (Fig. 1*H*). It was determined that 2 h of continuous rotation driven at the optimal imaging flow rate of 1 µL min^−1^ did not produce a significant change (97.5% to 94.0%, for N = 3 experimental repetitions, *P* = 0.1268, one-sided *t* test). Similarly, a higher flow rate of 5 µL min^−1^ produced negligible viability changes (95.0 ± 1.2%, N = 3). That flow rate value, however, was determined to be detrimental for imaging quality, as the five-fold increase in angular speed (~21 rpm) resulted in short exposure times (<2.5 ms for N = 1,200 slices), yielding insufficient photon counts to image the tested fluorophores. From these experiments, it was concluded that the flow rates relevant for imaging allowed for gentle handling of cells. This is further evidenced by estimation of the shear stress experienced by cells as they rotate inside droplets. If shear rate is taken to be proportional to the angular velocity (ω) of cells and η = 8.9 × 10^−4^ Pa·s, then the shear stress on cells can be approximated as $\tau_{s}∼\eta \omega$. For a single-cell rotation period of T= 5 to 8 s, this translates to a shear stress of $\tau_{s}∼$ 0.7 to 1.1 mPa, which is lower than arterial (~1.5 Pa) and venous (0.1 to 0.6 Pa) wall shear stresses (22, 23); and significantly less than the typical stress applied to cells going through a flow cytometry nozzle (~1 to 10 Pa) for a 70 µm nozzle operating at moderate flows of 10 to 100 µL·min^−1^, and a 20 µm cell) (24, 25). Consequently, high-throughput flow cytometry-based imaging techniques could result in higher shear stress, potentially reaching levels that could affect cell viability (26) or alter activation function in suspension cells like immune cells (27, 28), especially in the case of sensitive cell types or cells with outer membrane structures.

### Imaging Cellular and Subcellular 3D Features in Live Nonadherent Cells.

ADOPT can acquire immunofluorescent 3D volumetric data from suspended cells with isotropic resolution. We investigated its performance to quantify the presence of CD45 markers on the surface of K562 cells. Fig. 2*A* portrays the maximum intensity projection (MIP) of the fluorescent signal, while Fig. 2*B* takes an isosurface rendering of that 3D reconstruction using an isovalue of 0.32 with respect to the normalized fluorescence intensity in arbitrary units. These reconstructions evince the isotropic resolution available to OPT imaging. Similarly, the topography of smaller cells, such as primary T cells (activated and nonactivated) can be imaged with isotropic resolution at the diffraction limit, as estimated by the Rayleigh criterion (0.61 λ/NA = 244 nm for this fluorophore). For example, a rotation slice from a CD25+ T cell labeled with CD45 surface markers is shown in Fig. 2*C*, along with its corresponding isotropic surface topographic reconstruction in Fig. 2*D*.

We further demonstrated ADOPT can reconstruct intracellular structures like the DNA content of the nucleus. Diffraction-limited images of the nuclear content in live K562 cells, made visible through Hoechst 33342 staining, are displayed in Fig. 2*E*. A collection of N = 800 slices at varying angles (0.45°/slice, 150 fps, raw footage in Movie S3) were used to generate the 3D volumetric rendering in Fig. 2*F* (Movie S4), which features an estimated isotropic resolution of 216 nm. For all 3D surface models shown, shape morphometry can retrieve single-cell topographic, quantifiable data. The surface model in Fig. 2*G* presents the texture map of a K562 cell surface, where the color of each surface patch represents its distance from the 3D model average radius. Similarly, Fig. 2*H* provides a textural mapping of a highly concave K562 nuclear content distribution, featuring an enlarged nuclear groove. These measurements are therefore closely related to topographic and intracellular surface roughness and can potentially be used to compare single-cell metrics in a population manner for textural variations. Other single-cell morphometry parameters that can be calculated from our workflow include cell area, volume, and other morphological descriptors that offers enough discriminatory power, such as sphericity (29).

We further studied suspension cell nuclear content morphology by using a SPHARMs mathematical representation of the organelle surface data. The application of SPHARMs, an extension of Fourier techniques to three dimensions, is particularly advantageous for analyzing arbitrarily shaped but simply connected 3D closed biological structures with genus zero (surfaces without topological holes) in a quantifiable manner (30–32). We decided to apply this morphometry framework to extract nuclear feature details at coarser (low-frequency) and finer (high-frequency) spatial scales. This was motivated by the observation that ADOPT-collected 3D nuclear surface models possess high spatial frequency surface detail at the optical diffraction limit, but nonetheless have coarser shape features (e.g., large topographically concave nuclear grooves) that distinguish them from cell to cell. Application of the SPHARM algorithm leads to the measurement of SPHARM coefficients $c_{l}^{m}$ for each harmonic function $Y_{l}^{m}\left(\theta ,\phi \right)$ used in the expansion, up to a degree $l=L_{\max}$ (see *SI Appendix* for details). As seen in *SI Appendix*, Fig. S3*B*, the magnitude $\left|c_{\mathrm{kl}}^{m}\right|$ of each coefficient for each spatial coordinate, k=(x,y,z), was plotted against the canonical SPHARM numbers, in order of increasing azimuthal frequency (thus, a $L_{\max}$ degree reconstruction contains a total of ${\left(L_{\max}+1\right)}^{2}$ SPHARM numbers for each of the three spatial coordinates). A colormap in *SI Appendix*, Fig. S3*C* illustrates the magnitude of the SPHARM coefficients for each spatial coordinate and two different types of nuclear shapes (spherical and concave nuclei). This spectral decomposition allows identification of higher frequency components associated with the presence of a nuclear groove oriented along the *y* axis (Fig. 2*H*). Whereas a nearly spherically shaped K562 nuclear distribution displays similar x,y and z components with weak low-frequency components (*SI Appendix*, Fig. S3*C*), concave shapes possess stronger, nonuniform, coarse features required to fully recapitulate the nuclear groove topography.

Furthermore, Fig. 2*I* shows the effect of adding SPHARMs ($L_{\max}$ = 5, 10, and 20) to the reconstruction of a K562 nucleus surface model. The plot illustrates the presence of coarser topographical features like the concave nuclear groove at low frequencies, in agreement with the magnitude plots in *SI Appendix*, Fig. S3*C*. To automatically quantify the degree to which these coarse morphological features occur in single-cell K562 nuclei volumetric fluorescence data, we evaluated the concavity of each surface model via measurement of its local Gaussian curvature (33, 34), $G=\kappa_{1}\kappa_{2}$, where $\kappa_{i}$ represents the principal curvature components. We defined portions of the surface containing negative curvature to be concave, indicated as blue patches in Fig. 2*I*, and proceeded to compute the concave fraction of the nuclear surface area at each increasing level of detail (i.e., by increasing harmonics). We identified that low-frequency harmonics (3 through 8 in Fig. 2*J*) have the most significant impact on concavity when comparing spherical and concave nuclei (Fig. 2 *K* and *L*), suggesting morphological differences occur mostly on a coarse scale. Finally, we verified this mathematical approach to concave fraction measurement via SPHARM surface reconstruction was independent of cell orientation by analyzing cell shapes rotated at different random orientations (N = 583, *SI Appendix*, Fig. S4). Taken together, our isotropically resolved and orientation-independent analysis of 3D single-cell data at the diffraction limit provides a powerful framework for identifying morphometric structures across various cell feature length scales, while enabling morphometric quantification at the population level.

### ADOPT Performance Vs. Optical Sectioning Techniques.

**Fig. 3.**
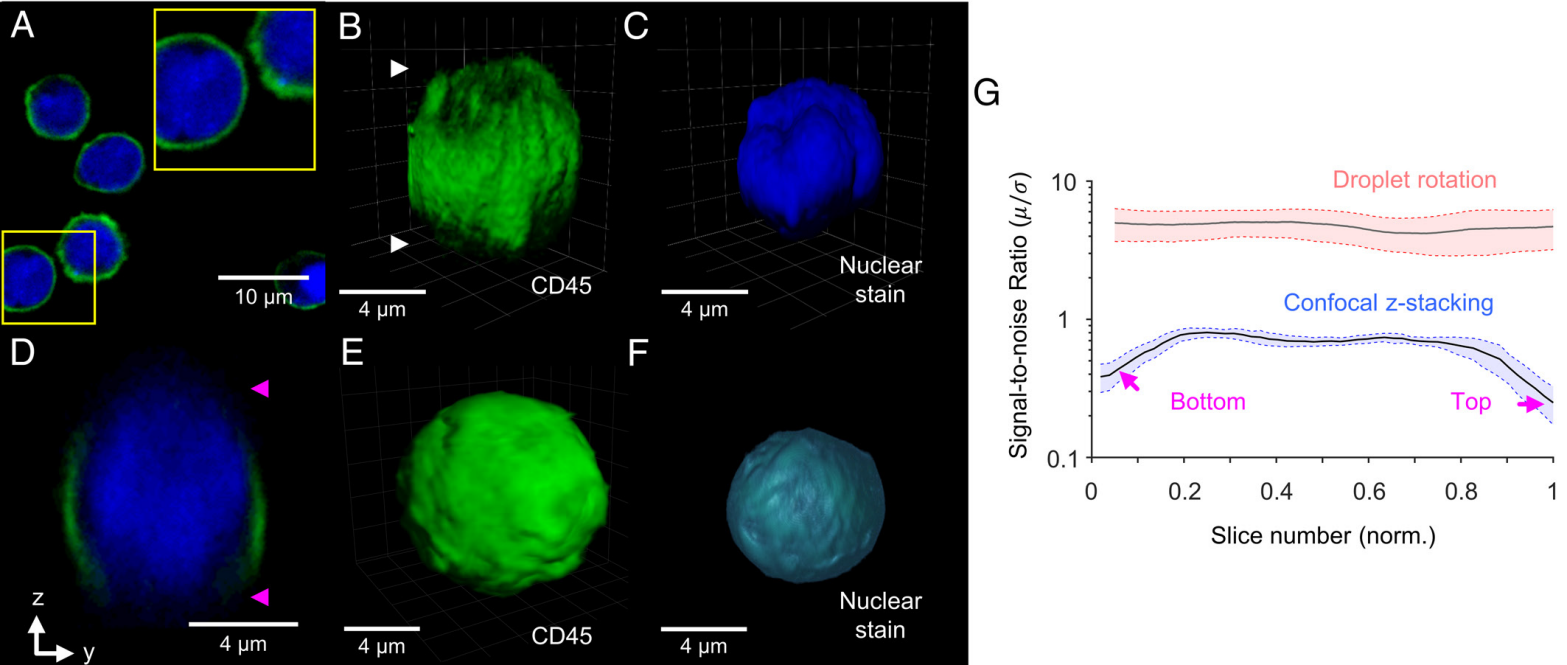
Tomographic imaging enabled by single-cell rotation acquisition mode enhances SNR characteristics. (*A*) CLSM optical section of naive T cells bound to the substrate, with their surface marked with anti-CD45-Alexa Fluor 488 and their nucleus with Hoechst 33342 dye. Blow-up shows the distribution of the nucleus with marked regions resembling lobes. (*B* and *C*) Z-stacking images of the surface (*B*) and nucleus (*C*) of T cells, showing the top and bottom artifacts in the reconstruction, as well as the reduced axial resolution. (*D*) An yz-plane cut shows the top and bottom optical sectioning artifacts. (*E* and *F*) ADOPT reconstructions of the surface and nucleus of an activated T cell. (*G*) Comparison of the SNR characteristics of ADOPT vs. CLSM as a function of the slice number (normalized by N = 61 slices for confocal and N = 500 slices for the droplet rotation case). Alexa Fluor 488 fluorophore was analyzed for (*G*) by triplicate in each case, and the frame grayscale values were normalized by the peak intensities in each case. Boundaries represent the average SNR values ± the SEM.

We compared the SNR characteristics of 3D images collected via CLSM and ADOPT. While CLSM remains a major workhorse in biomedical optical microscopy for its out-of-focus light rejection capabilities (e.g., Fig. 3*A*), it is susceptible to high depth-dependent degradation (4). We observed this artifact was present in z-stacked images of substrate-bound T cells labeled for surface and intracellular markers, as seen in Fig. 3 *B–D*. Conversely, T cells imaged with the ADOPT strategy were not visibly damaged by sectioning artifacts, as seen in Fig. 3 *E* and *F*. To quantify the degree to which the optical signal was degraded per collected slice, we computed its corresponding SNR, given by the ratio of signal average pixel intensity (μ) and SD (σ) of each slice. After normalizing for optical intensity, we observed ~3.2-fold decrease in SNR in confocal z-stacks when comparing the initial and final z-stack levels to mid-range slices (Fig. 3*G*). Cell-rotation slicing, in contrast, produces marginal changes (~1.2-fold) in SNR. Finally, we note that the volumetric reconstructions collected by CLSM had visible lower axial resolution owing to the PSF anisotropy. This observation was consistent with the calculated lateral and axial optical resolutions for the CLSM setup, which were 201 nm, and 1.003 µm, respectively.

Observing nonfixed immune suspension cells by z-stacking methods presents a challenge, considering the temporal resolution in techniques that perform pixel-wise scanning of biological samples. The presently tested CLSM equipment operated with pixel scanning dwell times of ~2 µs px^−1^, which even when evaluating low-resolution ROIs (256×256 to 512×512 for single-cell observation), brought the slice acquisition time close to the minutes scale for N = 100 stacks (~20 µm in *z*-direction). The effects of these prolonged capture times can be seen in the 3D live cell images and slice visualizations of *SI Appendix*, Fig. S5 *A*–*D*, where nonfixed T cells showed signs of both rotational and translational drift artifacts. Moreover, by performing multicell centroid tracking in time-lapse videos, we tracked the average drift of N = 94 unfixed cells (35) and plotted the results in *SI Appendix*, Fig. S5*E*. Interestingly, the observed tendency was that T cells drift monotonically overtime, reaching close to 0.8 ± 0.4 µm displacements in ~3.8 s. Conversely, cells that spin about their rotation axis driven by microvortices experience a deterministic, value-bounded average displacement (0.3 ± 0.1 µm) that is periodic in nature.

### Pixel-Wise Temporal Image Denoising Enabled by ADOPT.

**Fig. 4.**
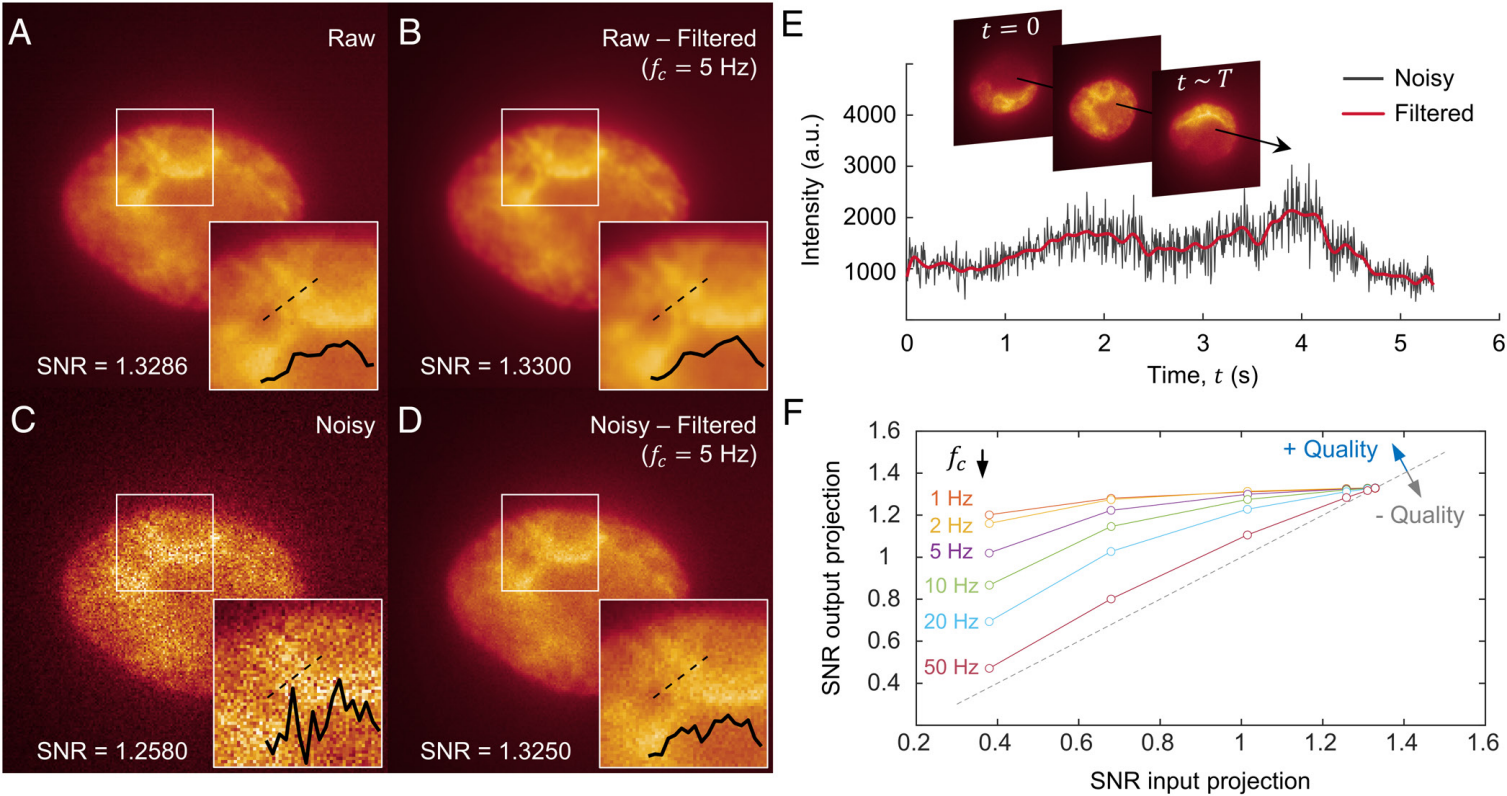
Single-cell rotation enables pixel-to-pixel temporal image denoising. (*A*) A 100X raw image (i.e., single-cell 2D projection) of a K562 cell nucleus stained with Hoechst 33342 dye. Insets show a blow-up of the boundary between two chromatin-rich structures and their corresponding line profiles. (*B*) Denoised image from (*A*), obtained by filtering the time-lapse signal of each pixel with a Butterworth filter (cutoff frequency, $f_{c}$ = 5 Hz). (*C*) Image in (*A*) with added Gaussian noise (SNR = 1.2580). (*D*) Denoised image from (*C*), following the same method as in (*B*). (*E*) Time-dependent signal coming from a single pixel (time evolution schematically emphasized by arrow). The plot demonstrates the use of a Butterworth filter to eliminate high-frequency components (low-pass filter). (*F*) Image quality improvement (measured as output SNR) as a function of the input noise level (SNR) and cutoff frequency $f_{c}$. The dashed line represents no net improvement (one-to-one SNR of input and output).

To improve the quality of reconstructions, we illustrated in Fig. 4 how the SNR of the collected time-lapse single-cell rotation frames can be greatly enhanced by using a pixel-to-pixel image restoration strategy. Considering that each set of frames containing the rotation of a cell is continually captured with a camera-based system (CMOS), the time-dependent photon influx information of each pixel can be restored by eliminating high-frequency components in the readout. This filtering process can be seen in Fig. 4 *A–D*, where the raw signal from a CMOS camera (Fig. 4*A*) is restored by applying a third-order, zero-phase low-pass Butterworth digital filter (Fig. 4*B*). In these figures, the nuclear content of a rotating K562 cell was visualized via live-cell staining. In Fig. 4*E*, the application of the described filter is demonstrated, showcasing the denoising of time-lapse information from a single pixel in Fig. 4*C*, collected over the full cell rotation duration (t=T). Furthermore, blow-up colormap plots in Fig. 4 *B* and *D* demonstrate the improved image quality achieved via filtering, which enhanced the visualization of the coarser chromatin boundaries present in the nucleus. This is further evinced by line profiles of the restored micrographs, which allowed estimation of the distance between these chromatin boundaries (~0.5 µm).

Fig. 4 *C* and *D* provides evidence that highly distorted images (SNR = 1.2580) can be restored to acceptable SNR levels that allow the distinction of coarser chromatin features. We validated the performance of our filtering algorithm by providing images with known SNR levels and found that signal improvement was largely dependent on the starting SNR values, as well as the low-pass filter cutoff frequency ($f_{c}$). By studying the effect of varying $f_{c}$ (Fig. 4*F*), we identified 5 Hz as providing a balance between enhanced SNR rates, while preserving high-frequency components in the image coming from the actual, time-dependent light readout. Further details on the noise characteristics of the CMOS-based sensor and filtering frequency criteria can be found in Supporting Information text.

### Primary T Cell Activation Phenotype and Nuclear Morphometry.

**Fig. 5.**
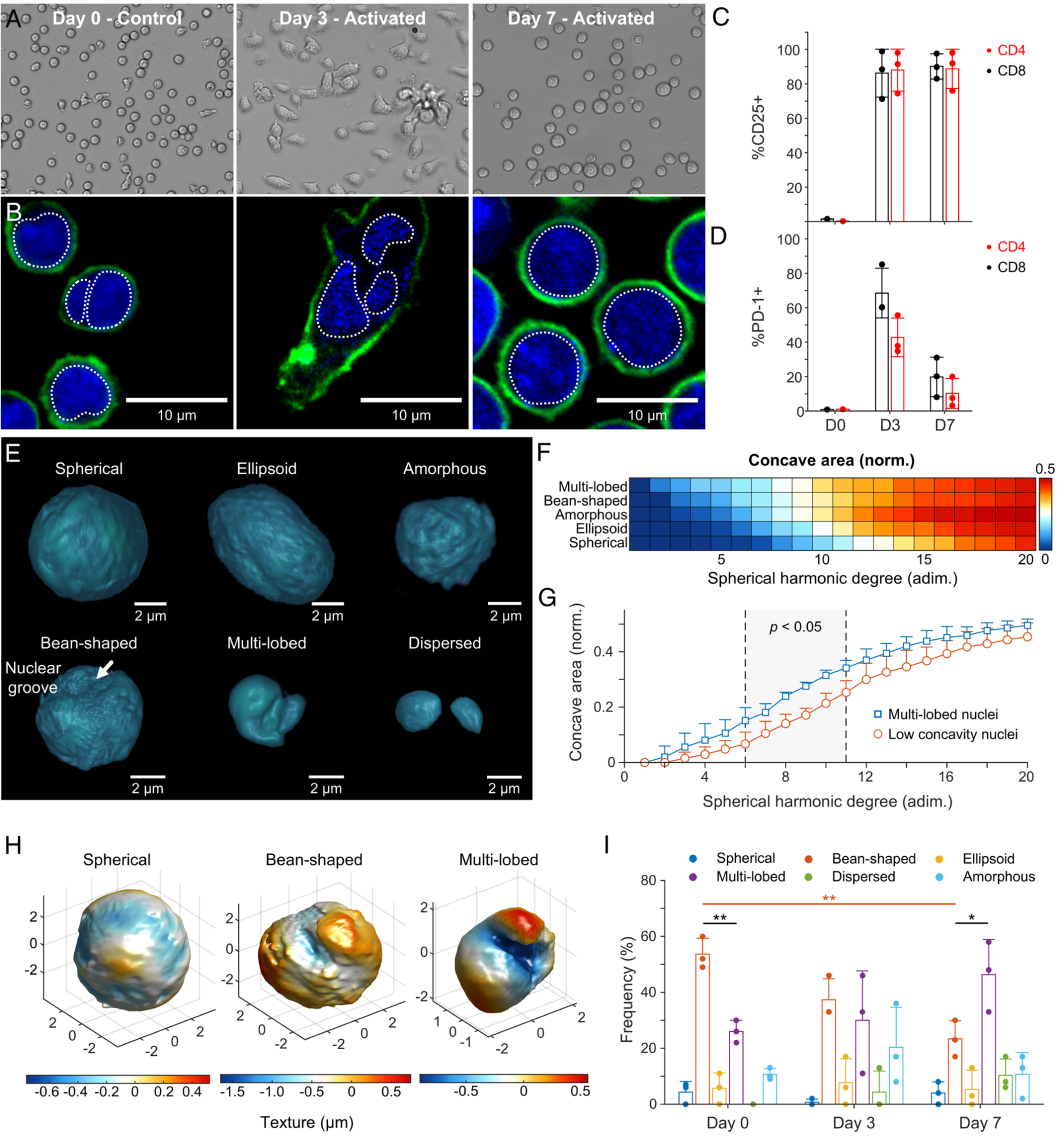
Primary T cell activation phenotype and nuclear morphometry via ADOPT. (*A*) Brightfield images of T cells as a function of the number of days after Dynabead-mediated activation. (*B*) Corresponding CLSM 2D optical sections, with cells stained for CD45 surface marker and nuclear content. (*C* and *D*) Percent of cells expressing CD25 and PD-1 markers for the N = 3 donors, plotted by days postactivation and classified by helper (CD4+) or cytotoxic (CD8+) phenotype. (*E*) Activated T cell nuclear content morphologies identified via ADOPT 3D reconstructions. (*F*) Colormap of the concave surface area fraction for simply connected T cell nuclear morphologies (excluding dispersed), plotted by increasing degree of SPHARM representation (i.e., increasing surface detail). (*G*) Surface concavity differences between the identified multilobed nuclei (N = 3), and low concavity morphologies (amorphous, ellipsoid, and spherical). (*H*) Three representative T cell nucleus morphologies of topological genus zero, illustrating their textural differences via a colormap. (*I*) Distribution of T cell nuclear morphology phenotypes, shown as a function of days after activation (N = 3 donors, two-tailed *t* test). Error bars throughout the figure represent mean values ± SD. In (*I*), *P < 0.05 and **P < 0.005.

Lymphocytes are particularly challenging to image without fixing protocols and thus are a fitting candidate for single-cell, 3D live isotropic imaging with ADOPT. We decided to image T cells in conditions where they would undergo visible phenotypic changes, such as in an immunological activation model. T cells were isolated from healthy donor whole blood samples (N = 3) using immunomagnetic negative selection. Cells were then imaged on days 0, 3, and 7 after their in vitro CD3/CD28 dynabead-mediated activation. We obtained 2D brightfield (Fig. 5*A*) and fluorescence optical sections (Fig. 5*B*) images of activated T cells via widefield and CLSM, respectively. We noted that control T cells display nearly circular or slightly bean-shaped nuclear distribution (Fig. 5*B*), consistent with previous reports (36). However, activated T cells exhibited an elongated phenotype, which was pronounced on day 3 following activation and stimulation with human recombinant IL-2. Morphological changes were further accompanied by an increased motility in T cells, as they surveyed the surface of well plates for contacting activator beads. Nuclear content in elongated T cells by day 3 was observed to decondense and acquire a multilobed or dispersed appearance, as seen in the CLSM fluorescence 2D sections of Fig. 5*B*.

Following human T cell activation and expansion (*SI Appendix*, Fig. S6*A*), cells experienced size growth during expansion as quantified in *SI Appendix*, Fig. S6*B*, from 5.8 ± 0.3 µm to 7.3 ± 0.9 µm, with a maximum size of nearly 11.1 µm by day 7. Other activation metrics were recorded, such as the percentage of T cells expressing CD25 and PD-1 markers (*SI Appendix*, Fig. S6 *C* and *D*), which were further classified by day and lymphocyte type (CD4+ or CD8+, Fig. 5 *C* and *D*). The expanded T cells showed a sustained expression of CD25 markers for the duration of the experiment (7 d), as expected from bead-mediated activation protocols (37). Additionally, expression of PD-1 was highest by day 3 (~65%) in cytotoxic CD8+ T cells, consistent with the expected high activation levels in healthy cells, which at day 3 postactivation display peak values according to manufacturing standards (38).

We applied ADOPT to the study of nuclear morphological changes that primary human T cells underwent upon anti-CD3/CD28 immunomagnetic bead-mediated activation. Owing to the 3D isotropic imaging capabilities of ADOPT, we identified six distinct nuclear morphologies by visual inspection of the isosurface renderings. These consisted of spherical, bean-shaped, ellipsoid, multilobed, dispersed, and amorphous nuclear shapes (Fig. 5*E*). To quantify these observed differences, we performed a SPHARM study on the 3D volumetric data, which allowed us to identify concavity changes as more harmonics were added to the analysis. We found that spherically distributed nuclear phenotypes increased in concavity only at higher harmonic frequency terms, whereas multilobed nuclei possessed marked concavity even at low harmonic numbers (Fig. 5*F*). It was also found that both multilobed and spherically distributed nuclei tend to a concave area coverage of 50% at higher frequencies, which can be explained by observing that anywhere locally on the nuclear surface models, there is an equal chance of finding concave and convex details due to the increasing complex surface roughness. Our method therefore allowed us to quantitatively find at what level of detail (harmonic) one can expect the most statistically significant morphological differences between two given nuclear phenotypes (Fig. 5*G*). These results are consistent with the low colormap texture values observed at the nuclear groove when comparing different T cell nuclei shapes (Fig. 5*H*).

In Fig. 5*I*, an analysis of activated T cells across 7 d for N = 3 donors illustrated an activation-dependent distribution of the identified phenotypic nuclear morphologies. We observed that most naive T cells had a nearly circular bean-shape distribution, which after immunological activation markedly switched to a predominantly multilobed population. This was evinced by the significant change in the amounts of bean-shaped nuclei from day 0 to 7 (*P* < 0.005) and the relative amount of bean-shaped to multilobed nuclei (*P* < 0.05) according to a two-sided *t* test. Although other morphologies were qualitatively identified, their frequency was low in comparison. While cells were analyzed in their suspended phenotype, the multilobed nature of the nuclear content identified in substrate-attached T cells seen in Fig. 5*B* (day 3) was preserved. This suggests that the dispersed nucleus nature of motile, activated T cells might require additional time to return to a more condensed state.

## Discussion

Understanding T cell activation dynamics and their concomitant structural phenotypic changes is crucial for elucidating their adaptive immune responses and effector functions. Morphological alterations, such as size growth and cytoskeletal rearrangement (39), prepare cells for division and immunological synapse formation (2). We showed that T cell volumetric nuclear content undergoes significant reconfiguration in normal culturing conditions for immunological activation and that these morphological changes persist after cells return to their suspended phenotype (Fig. 5 *E* and *I*). ADOPT is able to showcase 3D immunofluorescent and isotropically resolved reconstructions of live primary T cells fully suspended in liquid, detailing surface and nuclear structures at the optical diffraction limit (Fig. 5 *E* and *H*). These cells, being at the smaller size spectrum of leukocytes (5 to 7 µm), present a technical imaging challenge in their nonadherent state, drifting at rates capable of introducing significant artifacts, even on the timescale of seconds (0.2 ± 0.1 µm s^−1^, *SI Appendix*, Fig. S5*E*). While attachment of T cells to coverslips using fibronectin produces excellent imaging results using high spatiotemporal resolution optical systems (2), it can also influence the microenvironment in which cells operate, as glycoproteins can affect cell movement and generate costimulatory signals that activate degranulation (40, 41). These cues could therefore introduce extraneous integrin-mediated interactions that trigger cytoskeletal remodeling, cell motility, or receptor signal modulation (42). In contrast, our device overcomes these limitations without the need for surface treatments or cell fixing, ensuring compatibility with specific liquid media required by suspended cells. ADOPT therefore bridges a technological gap in live single-cell biomedical optics by leveraging the cell manipulation capabilities of intradroplet microvortices, which were shown to produce periodic and stably bounded cell rotation (*SI Appendix*, Fig. S5*E*).

Varied nonadherent cell nuclear structures were identified in this work (Figs. 2 and 5*E*), raising significant implications that warrant further investigation. Previous research quantified diverse 3D nuclear shapes not readily apparent in 2D cross-sections in normal, metastatic, and fibrocystic cells (29). In that study, a prevalent morphology termed the “mushroom cap” nucleus shape was found. This form resembles the highly concave nuclear distributions observed in our experiments with K562 cells, a human immortalized myelogenous leukemia cell line (Fig. 2 *F* and *H*). Myeloid and cancerous cells typically exhibit bean-shaped, lobulated, or segmented nuclei, which have been posited to confer morphological flexibility (36). Indeed, the relationship between nuclear morphology and the coarser features of chromatin structural configuration has been linked to cancerous states (43). Chromosome territories are stratified, with transcriptionally silent domains often located in the nuclear periphery, and content relocation occasionally varying with gene activation (44). Our results demonstrate that ADOPT-driven isotropic collection of 3D cell nuclear data can be used to quantify these coarser features via SPHARM analysis, which allows for a registration and orientation-independent concavity analysis between surface models. Recognizing the critical role of genome architecture in regulating transcription, DNA damage/repair, aging, and disease (45), ADOPT is poised to contribute to large-scale, multidimensional data-driven identification of morphometrically associated cell states and events.

Nuclear morphologies of T cells in different functional states have similarly been subjected to 3D volumetric analysis. Our combined ADOPT and SPHARM methodology provides an isotropic and orientation-independent morphometry analysis of suspended T cell nuclei detailing activation-driven surface concavity changes at the optical diffraction limit. While lymphocytes are generally recognized to have round, uniform, and relatively rigid nuclear shapes that preserve genomic DNA integrity during circulation (36), events such as immunological activation can lead to actin-mediated elastic forces that deform their nucleus (46). T cells undergo shape changes in physiological conditions, resulting in actin-dependent nuclear elongation that correlates with gene expression, Erk and NF-κB signaling to the nucleus, and the expression of activation markers such as CD69 (47). After immunological activation in vivo, T cells exit lymph nodes, enter the bloodstream, and subsequently reenter nonlymphoid organs, engaging in transendothelial migration to execute their effector functions (48). Transendothelial migration critically requires motor proteins, such as Myosin-IIA, to complete the movement of their rigid nucleus through endothelial junctions (48). Taken together, the results in Fig. 5 seem to support the hypothesis that a dispersed nucleus content in day 3, with its concomitant CD25 and PD-1 expression increases, can prepare T cells for the functional challenge of undergoing actin-driven phenotypic changes.

Other functional states in T cells associated with nuclear structure include the chromatin openness of specific genomic locations, such as the PD-1 genes. This chromatin openness has been shown to have predictive power for the effectiveness of anti-PD-1 immune checkpoint therapies in gastric cancer patients (49). Additionally, the study of other diseases, such as Nodular lymphocyte-predominant Hodgkin lymphoma (NLPHL) and T cell/histiocyte-rich large B cell lymphoma (THRLBCL), further emphasizes the importance of nuclear 3D analyses in activated T cells (50). Particularly, whereas 2D optical sections of T cell nuclei showed no significant enlargements, 3D volumetric measurements were necessary to show the significant differences between NLPHL and THRLBCL. Furthermore, recent studies on different CD8+ T cells point to the potential of 3D morphological features, such as volume, dry mass, refractive index, and spatial distributions as biomarkers for sepsis (51). We thereby anticipate that ADOPT quantification of multiparametric, immunofluorescence volumetric data of cells could not only potentially assess the functional state of T cells through nuclear measurements, but also help identify diseased or exhausted states of cells.

In Table 1, we provide a comparison of ADOPT with other 3D imaging technologies used for nonadherent cells, highlighting key features critical for single-cell analysis. Minute-frequency 3D imaging tomographic approaches quantifying optical phase changes in single live cells constitute important complementary technologies to ADOPT (16). While these methods possess nonisotropic resolutions (0.72 μm and 4.26 μm for the lateral and axial resolutions) (16), they have shown scalability and label-free capacities, whereas ADOPT in its current rendition only demonstrates an epifluorescence widefield microscopy modality. The isotropic morphometry and specificity gained from chemical labeling are therefore factors to be thoroughly considered in experimental design—especially in light of the single-cell analysis advantages that fluorescence-activated sorting could bring when combined with droplet sorting preparative procedures (52). Other label-free optical methods have further demonstrated isotropically resolved imaging based on tomographic molds for optical trapping (53), with resolution at the diffraction limit, but are incapable of suspended cell phenotypic imaging or multiplexed, chemically specific signal acquisition. Further limitations of ADOPT include its use of fluorescence OPT mode, which is prone to rotation artifacts far from the object’s center of rotation (54). PSF-aware reconstruction algorithms or focal plane scanning strategies (55) can alleviate this issue, albeit at increased acquisition times.

**Table 1.** Suspension cell 3D imaging techniques

Imaging technique	Imaging mode	Imaging speed	Scalability	Isotropy	Resolution	Complexity	Fixation/substrate treatment	Shear forces on cells	Cell diffusive cross talk	Media restrictions	Ref.
ADOPT	Epifluorescence	High	High	Yes	High	Low	No	Very low	No	No	This work.
CLSM	Confocal	Low	Very low	No	Very high	Very high	Yes	N/A	Yes	No	(52)
Spinning disk confocal microscopy (SDCM)	Confocal	High	Low	No	Very high	Very high	Yes	N/A	Yes	No	(53)
LLSM	Light-sheet	High	Low	No	Very high	Very high	Yes	N/A	Yes	No	(2)
Structured-illumination microscopy (SIM)	SIM	Moderate	Low	No	Super resolution	Very high	Yes	N/A	Yes	No	(54)
Multiphoton microscopy	Multiphoton	Low	Very low	No	Very high	Very high	Yes	N/A	Yes	No	(55)
Live cell computed tomography (LCCT)	Epifluorescence	High	Low	Yes	High	Moderate	No	Very low	Yes	Electrorotation compatible media	(6)
Highly inclined and laminated optical LCCT	Light-sheet	High	Low	Yes	High	Moderate	No	Very low	Yes	Electrorotation compatible media	(10)
Light-field flow cytometry	Light-field	Very high	Very high	No	Moderate	Moderate	No	High	Yes	No	(8)
Tomographic imaging flow cytometry	Varies	High	High	No	Moderate	High	No	Varies	Yes	No	(7)
Refractive-index (RI) tomographic imaging	Microtomography (No fluorescence)	High	Moderate	Yes	High	High	No	N/A	Yes	No	(56)

The simplicity of the wide-field and camera-based acquisition scheme utilized by ADOPT makes it an attractive 3D single-cell analysis platform. ADOPT is expected to be incorporated into the droplet microfluidic toolbox by providing single-cell 3D spatial information that can be correlated with cell state and function. Because comprehensive immunophenotyping of T cell state necessitates both the study of labeled surface antigens and secreted cytokines (60, 61), coencapsulation of single cells with anti-cytokine detection probes in the ADOPT workflow could be used to eliminate protein diffusive cross talk typically seen in bulk assays (62, 63) and facilitate the correlation of 3D single-cell morphometric and secretomic information. Droplets can also be extracted and merged for analysis outside the device after on-chip examination via clone or bulk recovery strategies (63, 64), providing further flexibility in experimental workflows. Moreover, the controlled shear stress exerted on cells as they are rotating was shown and could be utilized for precise mechanical stimulation on single cells to study its biomechanical properties. Finally, single-cell droplet sorting technologies could further provide an opportunity for high-throughput, multiplexed analysis of specific cell subtypes. These benefits render ADOPT modular and adaptable to preceding steps in droplet preparation, thus broadening the scope of microfluidic tools for multidimensional single-cell analysis in biomedical applications.

## Materials and Methods

### Microfluidic Chip Fabrication.

Arrays of droplet traps, made from polydimethylsiloxane (PDMS), were manufactured through a two-layer standard soft-lithography process. For the photolithography steps, a first 40 µm thick layer of SU-8 3025 (Kayaku, MA) was spin-coated on a clean Si wafer (100) at 500 rpm for 10 s at an acceleration of 100 rpm s^−1^, followed by 2,000 rpm for 30 s at an acceleration of 300 rpm s^−1^. This was followed by soft baking at 95 °C for 15 min, UV exposure (365 nm) at a dose of 150 mJ cm^−2^, and a two-step post-exposure bake (PEB) of 1 min at 65 °C and 3 min at 95 °C. A second 30 µm thick layer was thereafter coated at 2,500 rpm following the aforementioned acceleration procedure. Soft bake was then carried out at 95 °C for 10 min, a second UV exposure at ~125 mJ cm^−2^, and the previously mentioned PEB steps. The SU-8 material was developed for 7 min, with the last minute aided by sonication to completely etch the inverted microwell circular structures (traps).

After photolithography, Sylgard 184 monomers and silicone elastomer curing agent (Dow Corning) were mixed at a 10:1 ratio and cast into the SU-8 microfluidic master structures. The liquid mixture was degassed for 1 to 2 h in a desiccator and then allowed to partially cure overnight at room temperature. Subsequently, the mixture was heated to 65 °C for 24 to 48 h in a convection oven. The solidified PDMS material was removed from the molds, hole-punched, and any debris was cleared using scotch tape. Finally, the PDMS device was plasma treated (Harrick Plasma) for 1 min and bonded to clean, thin glass slides (170 µm thick).

### Droplet Encapsulation of Cells.

A previously described microfluidic device with 40 µm in height was employed for the generation of droplets containing suspension cells (19). Specifically, the continuous phase consisted of 2% (w/v) 008-FluoroSurfactant in HFE 7500 oil (Ran Biotechnologies), while the dispersed phase comprised 1x DPBS, 16% Optiprep (STEMCELL Technologies, Canada) along with the suspended cells. Cells were washed and prepared to a concentration of 10^7^ cells mL^−1^ in the dispersed phase. For encapsulated dead cell exclusion and viability analysis, propidium iodide solution (BioLegend) was added to the dispersed phase at a concentration of 2 µL per 100 µL of cell solution (with 1 × 10^6^ cells mL^−1^), and incubated at 4 °C for 15 min prior to imaging.

Picoliter-sized droplets were generated using two Flow EZ pressure control modules (LU-FEZ-7000, Fluigent). First, microdevices were primed with the pressure pump delivering the continuous phase at a pressure of 115 mbar. Once devices were primed, the second module was set to 120 to 140 mbar, which resulted in squeezed cell-laden droplets with a diameter of 65 to 71 µm, as seen from 2D images from a conventional widefield inverted microscope at 10× magnification (Olympus IX83). Specifically, smaller droplets (65 µm diameter from circular view) were optimal for imaging naive T cells (5 to 7 µm), whereas bigger droplets (71 µm) performed best for activated T cells (6 to 12 µm) and K562 cells (10 to 20 µm). Finally, droplets were collected for a duration of 30 min and subsequently stored in 1.5 mL Eppendorf tubes (Eppendorf, Germany).

### Droplet Entrapment Using Inverted Microwell Array.

Prior to the droplet loading experiment, devices were placed in vacuum for 5 min to prevent the formation of any bubbles during sample input and thereafter primed with continuous phase oil. After droplet generation and trap device priming, 10 µL of cell-laden droplets were pipetted into the inlet reservoir of the microfluidic trap array device. A pipette tip (200 µL) inserted into the inlet of the device acted as a reservoir for droplets (*SI Appendix*, Fig. S1). Continuous phase oil was then perfused into the chip using a syringe pump in withdrawal mode at 4 µL min^−1^ (Pump 11 Elite, Harvard Apparatus), which eventually resulted in droplets loading into the array. Once all traps were occupied by droplets, the flow rate was set to 1 to 2 µL min^−1^ to perform cell rotation for 3D live cell imaging. Cell rotation rates were also characterized using a custom-made routine (MATLAB) as a function of the applied continuous phase flow rate (1 to 10 µL min^−1^).

Fluorescein isothiocyanate-dextran (250,000 MW, Sigma Aldrich) was used to enhance droplet visualization and for droplet encapsulation efficiency determination. Briefly, the total number of traps occupied by droplets was counted and evaluated versus the number of available traps (n = 576); the efficiency of single droplet occupation was similarly determined by inspection of the microfluidic array. Suspension cells (K562 cell line) were stained with PE anti-human CD45 antibody (BioLegend) for ease of visualization.

### ADOPT Operation.

After droplet loading into microfluidic traps, an oil flow rate of 1 to 2 µL min^−1^ was used to produce cell rotation rates of 4.2 to 8.4 rpm. This allowed acquisition of N = 800 to 1,200 slices per cell at a 6.67 ms exposure (150 fps) which were obtained using an Olympus UPlanFL N 100× oil immersion objective (NA = 1.3) mounted on an Olympus IX51 epifluorescence microscope (Olympus). Our OPT routine is based on a previously reported direct inversion algorithm which helped reduce noise from out-of-focus light (55, 65). Furthermore, monochrome 204×204 px 16-bit frames were collected using a CS505MU Kiralux 5.0 MP monochrome CMOS camera (Thorlabs) with a 3 × 3 binning scheme to increase SNR. These settings resulted in an xy image resolution of 79 nm px^−1^.

Prior to building the 3D volumetric reconstructions from CMOS footage, pixel-to-pixel image restoration was carried out by performing signal filtering. For each set of time-lapse frames containing a full rotation of a cell, the time-dependent signal of each pixel was filtered using a third-order, zero-phase Butterworth digital filter with a defined cutoff frequency, $f_{c}$ (1 to 50 Hz). After performing SNR optimization (Fig. 4*F*), $f_{c}$ = 5 Hz was chosen to filter all collected time-lapse frames in this study, unless otherwise specified.

To visualize cell surface topography, K562 cells and T cells were stained with anti-CD45 Alexa Fluor 488 (HI30, Invitrogen). Intracellular imaging of cell nuclei was performed using NucBlue™ (Hoechst 33342 dye, Invitrogen) by following the manufacturer’s instructions. For T cell activation experiments, the nuclear content morphometry of 100 cells was assessed on days 0, 3, and 7, and classified according to its corresponding morphology (spherical, bean-shaped, ellipsoid, multilobed, dispersed, or amorphous).

### Cell Culture.

K562 cells obtained from the American Type Culture Collection (ATCC) were cultured in IMDM medium (Gibco) supplemented with 10% heat-inactivated fetal bovine serum (HI-FBS; Gibco) and 100 U mL-1 penicillin–streptomycin (Gibco). Cells were consistently maintained in a humidified incubator at 37 °C with 5% CO_2_ and passaged every 2 to 3 d using standard procedures. Cell encapsulation in droplets and trapping experiments were conducted on the same day that cells were subcultured.

### Primary T Cell Isolation, Activation, and Expansion.

Deidentified healthy donor blood samples were procured from the Institute for Clinical and Translational Science (ICTS) at the University of California, Irvine. All blood samples used in this study were collected following ethical guidelines and with the necessary informed consent to ensure patient confidentiality. Within less than 3 h of blood collection, primary human T cells were obtained via standard immunomagnetic bead-based negative selection. Direct Human T cells isolation kits (EasySep™, STEMCELL Technologies) were used according to the manufacturer’s instructions.

Primary human T cells underwent activation and expansion using standard bead-based protocols. Initially, freshly isolated T cells were seeded at a concentration of 0.2 to 0.5 × 10^6^ cells mL^−1^ in 24-well plates with 1 mL of RPMI 1640 medium, ATCC modification (Gibco). The medium was supplemented with 10% HI-FBS (Gibco), 2 mM L-glutamine, 1 mM sodium pyruvate, 0.1 mM nonessential amino acids, 10 mM HEPES, and 100 U mL^−1^ of penicillin-streptomycin (Gibco). To activate and expand T cells, media-washed human activator CD3/CD28 Dynabeads (Gibco) were added at a 1:1 cell-to-bead ratio, and the media further supplemented with 30 U mL^−1^ of human recombinant IL-2 protein (STEMCELL Technologies). T cells were incubated in a humidified incubator with 5% CO_2_ at 37 °C and were split to 0.5 × 10^6^ cells mL^−1^ when their concentration surpassed 2.5 × 10^6^ cells per well or when medium became depleted (turning yellow in color). Expansion fold numbers were recorded on days 0, 3, and 7.

### T Cell Immunophenotyping.

Human T cells were concurrently stained with anti-CD4 Pacific Blue (OKT-4, BioLegend), anti-CD8a APC (RPA-T8, Invitrogen), and either anti-CD25 FITC (BC-69, BioLegend) or anti-PD-1 FITC (MIH4, Invitrogen) to determine their levels of activation during expansion experiments (days 0 to 7). Standard washing and incubation procedures for flow cytometry were followed from BioLegend. Dye 7-AAD (BioLegend) was used for dead cell exclusion from the analysis. A NovoCyte 3000 flow cytometer (ACEA Biosciences, CA) was used to analyze the cell suspension samples. Single-color stained cells were used to determine compensation levels, whereas positive and negative control samples were used to determine gating levels for quantification.

### Unfixed T Cell Drift Tracking.

The movement of T cells inside 24-well plates without any treatment or fixing was tracked to quantify drift in their suspended phenotype. Conventional brightfield images were collected in widefield mode using an Olympus IX51 microscope equipped with a 10x air objective. Videos were collected at 50 fps, with 1224×1080 resolution at 2 × 2 binning using a CS505MU Kiralux 5.0 MP monochrome CMOS camera (Thorlabs). A multiobject tracking algorithm built in Matlab was used to create a population metric of the time-lapse drift of cells (35). The total cell displacement, $r_{c}=\sqrt{x_{c}^{2}+y_{c}^{2}}$, where ($x_{c},y_{c}$) was the position of cells in each individual frame, was calculated for a total of N = 94 cells.

### Immunofluorescent Confocal Imaging.

Confocal imaging of substrate unbound and bound human T cells on days 0, 3, and 7 was performed using an Olympus FluoView FV3000RS CLSM. Briefly, cells were simultaneously stained with Hoechst 33342 dye and anti-CD45-Alexa Fluor 488 to visualize surface and intracellular features and excited using a one-way sequential line scan at 350 nm and 488 nm with a pinhole size of 167 µm and a scan speed of 2 µs px^−1^. In addition, nonactivated T cells were imaged for control purposes. Cells were observed using an Olympus UPLSAPO 40× silicone oil immersion objective (NA = 1.25). The obtained pixel resolutions were 78 nm px^−1^ in the xy direction, and 200 nm px^−1^ in the z direction. A z-step size of 200 nm was used to generate the 3D reconstructions of live fixed and unfixed T cells. With the described settings, calculated optical lateral resolutions were 201 nm, and 1.003 µm for the axial direction.

## Supplementary Material

Appendix 01 (PDF)

Movie S1. Brightfield footage of a rotating K562 cell trapped inside droplet under 2 μL min^−1^ external oil phase flow rate. Scale bar = 20 μm.

Movie S2. Brightfield footage of a rotating K562 cell trapped inside droplet under 10 μL min^−1^ external oil phase flow rate. Scale bar = 20 μm.

Movie S3. A rotating K562 cell trapped inside droplet with its nucleus stained using Hoechst 33342. Video was captured at 150 fps using a DAPI filter in a conventional widefield epifluorescence microscope at 100x (NA=1.3). Scale bar = 4 μm.

Movie S4. Reconstructed 3D surface model obtained via Optical Projection Tomography of raw footage in Movie S3. Surface was constructed via an isosurface rendering of the 3D volumetric fluorescence intensity profile. Scale bar = 4 μm.

## Data Availability

The custom scripts used for analysis are available at GitHub (https://github.com/braulio-cardenas/BioMiNT-ADOPT) (66). Data files, including MATLAB .mat files and raw images, are available in a Figshare repository (https://doi.org/10.6084/m9.figshare.27048832.v2) (67). All other necessary code used in producing the analysis for this work was performed using freely available algorithms written by other groups, including i) the SPHARM-MAT analysis tool [https://www.med.upenn.edu/shenlab/spharm-mat.html, (30, 31)]; ii) the splineradon routine [https://web.archive.org/web/20180615063712/http://sybil.ece.ucsb.edu/pages/splineradon/splineradon.html, (65)]; iii) the PSF generator Java software (https://bigwww.epfl.ch/algorithms/psfgenerator/#download); iv) the FPS-OPT reconstruction algorithm [https://web.archive.org/web/20230222233032/http://sybil.ece.ucsb.edu/pages/fpsopt/, (55)]; and v) the TracTrac MATLAB source code [https://github.com/jorishey1234/tractrac, (35)].
