# Supplementary material for: Three-dimensional isotropic imaging of live suspension cells enabled by droplet microvortices
Source: Proc Natl Acad Sci U S A. 2024 Oct 22;121(44):e2408567121. doi: 10.1073/pnas.2408567121 (PMC11536124; doi:10.1073/pnas.2408567121)
Supplement: Supplementary file 1 — Appendix 01 (PDF) [file pnas.2408567121.sapp.pdf]

PNAS

Supporting Information for
Three-dimensional Isotropic Imaging of Live Suspension Cells Enabled by Droplet Microvortices

Braulio Cardenas-Benitez[a,b], Richard Hurtado[a,b], Xuhao Luo[a,b] and Abraham P. Lee[a,b,c,*]

[a] Department of Biomedical Engineering, University of California, Irvine; Irvine, 92697, United States of America.

[b] Center for Advanced Design & Manufacturing of Integrated Microfluidics (CADMIM), University of California, Irvine; Irvine, 92697, United States of America.

[c] Department of Mechanical & Aerospace Engineering, University of California, Irvine; Irvine, 92697, United States of America.

*Corresponding author: Abraham Phillip Lee

Email: aplee@uci.edu

This PDF file includes:

Supporting text
Figures S1 to S6
Legends for Movies S1 to S4
SI References

Other supporting materials for this manuscript include the following:

Movies S1 to S4

Supporting Information Text

Theoretical flow recirculation timescale in droplets

The simplest theoretical model that captures the minimal elements to represent inner droplet flow in 3D is that of an inner phase static spherical droplet of radius R_0 at the origin, with dynamic viscosity η_i, submerged in a continuous outer phase of dynamic viscosity η_o that flows at uniform velocity U_0 far from the fixed spherical droplet. Under these conditions, the droplet will experience shear-stress that results in inner ($\mathbf{u}^i$) and outer ($\mathbf{u}^o$) flow velocity fields. Assuming a laminar, inertia-less flow and neglecting surface tension gradients, both fields admit analytical representations that are known as Hadamard-Rybczynski velocity fields (*1*). These velocity fields, although idealized and incomplete for fully recapitulating the 3D configuration of trapped droplets, describe some of the functional relationships between quantities of interest with the physical parameters of the problem, and thus facilitates the study of inner droplet flow. Fig. S2 illustrates the Hadamard-Rybczynski velocity field streamlines as the outer phase with velocity $-U_0\,\hat{\mathbf{z}}$ flows past the sphere. Under these conditions, the inside velocity field can be fully represented with radial and angular components in spherical coordinates, $\mathbf{u}^i = \left(u^i_\rho, u^i_\theta\right)$:

$$u^i_\rho(\rho,\theta) = \frac{R_0}{\tau}\cos\theta\left[\left(\frac{\rho}{R_0}\right)^2 - 1\right], \tag{S1a}$$

$$u^i_\theta(r,\theta) = \frac{R_0}{\tau}\sin\theta\left[2\left(\frac{\rho}{R_0}\right)^2 - 1\right], \tag{S1b}$$

where a characteristic timescale τ has been identified:

$$\tau = \frac{2R_0}{U_0}\,(1+q), \quad q = \frac{\eta_i}{\eta_o}. \tag{S2}$$

Hadamard-Rybczynski velocity streamlines inside the inner phase describe a family of Bernoullian quartic curves which admit the following parametric equation representation:

$$\frac{\rho_0}{\rho}\sqrt{\frac{\rho_0^2 - R_0^2}{\rho^2 - R_0^2}} = \sin(\theta), \tag{S3}$$

where ρ_0 is the starting radial defining a specific closed quartic curve (Fig. S2). Ideally small particles recirculate these closed pathlines, thereby describing a periodic motion on both $\rho(t)$ and $\theta(t)$ coordinates. This orbiting period, T_{orbit}, can be obtained by solving a system of ordinary differential equations that are defined by the radial and angular components of the velocity field (Eqs. S1a,b). We found that the solution to this system of nonlinear differential equations can be written in the implicit form:

$$t - t_0 = \tau\int_{\rho_0}^{\rho}\frac{R_0\rho' d\rho'}{\sqrt{({\rho'}^2 - R_0^2)({\rho'}^2 - \rho_0^2)({\rho'}^2 + \rho_0^2 - R_0^2)}}, \tag{S4}$$

where $\rho(t_0) = \rho_0$ set as the start point of the particle path, and the time constant τ is given by Eq. (S2). From Eq. (S4), the recirculation time or cell/particle orbiting period can be readily computed as:

$$T_{orbit} = \frac{2\tau}{\sqrt{(\rho_0/R_0)^2 - 1}}\left[K\left(\frac{\rho_0^2}{R_0^2 - \rho_0^2}\right) - F\left(\phi, \frac{\rho_0^2}{R_0^2 - \rho_0^2}\right)\right] = \tau\, f(\rho_0, R_0), \tag{S5}$$

where ρ_0 is the vortex starting point, $K(m)$ and $F(\phi, m)$ are the complete and incomplete elliptic integrals of the first kind, respectively, with $\phi = \sin^{-1}[({R_0}^2 - {\rho_0}^2)/{\rho_0}^2]$ and $m = \frac{\rho_0^2}{R_0^2 - \rho_0^2}$. Although being an implicit solution to the particle equations of motion, Eq. (S5) can be directly used to compute particle trajectories.

Because $T_{orbit} \sim \tau$ for a given recirculation streamline and the outer flow velocity magnitude is proportional to the applied flowrate ($U_0 \sim \alpha Q$), the angular velocity of a recirculation element is given by:

$$\omega = 2\pi/\tau = \frac{2\pi}{\frac{2R_0}{U_0}(1+q)} = \frac{\pi U_0}{R_0\left(1+\frac{\eta_i}{\eta_o}\right)}$$

$$\omega(Q) = \frac{\pi\alpha Q}{R_0(1+\eta_i/\eta_o)}\ . \qquad \text{(S6)}$$

Noise sources in the CMOS-based detection system

In our detection system, high-frequency noise primarily originates from the camera-based detection, specifically from the complementary metal-oxide semiconductor (CMOS). That readout noise frequency significantly exceeds that of the temporal photon flux provided by the rotating cell, which has frequency in the order of $f_0 \sim 1/T$, where T is the cell rotation period.

We provide a noise analysis for the CMOS system, based on the work of Mandracchia et al. (*2*). In CMOS sensors, different electronic elements convert impinging photons (P) into photoelectrons (S) and thereafter into an intensity value or digital number (DN), for each pixel in the array that acts as a detector (*2*). Each element involved in the transduction process introduces uncertainty, which can be modeled via the following equation:

$$DN(p,t) = g(p) \cdot S'(p,t) + o(p) \quad \text{(S7)}$$

where $DN(p,t)$ represents the digital number (grayscale value of the camera) readout at pixel p at timepoint t. In this equation, the fixed gain in DN/e^-units at pixel p is given by $g(p)$, and the offset $o(p)$ is given in DN units. The number of electrons $S'(p,t)$ measured at a given pixel p and timepoint t is the compound effect of different statistical processes that introduce the noise, and is described by the following equation:

$$S'(p,t) = P(S;p,t) + P(I_D\tau;p) + \mathcal{g}(0,\sigma_R^2;p) + \mathcal{g}(0,\sigma_{FPN}^2;p,t) \quad \text{(S8)}$$

where $P(\cdot)$ is the Poisson distribution, $\mathcal{g}(\cdot)$ the heteroskedastic Gaussian distribution, I_D is the dark current, τ the exposure time, σ_R^2 is the electronic readout noise variance, and σ_{FPN}^2 the fixed pattern noise variance. In Eq. (S8), the first term is due to photon shot noise, the second term to dark shot noise, the third to electronic read noise, and the last to fixed pattern noise. Short exposure times (<1 s) lead to the safe assumption that dark current can be neglected (*3*). Following the analysis of Mandracchia et al. (*3*), the Poisson distribution can be replaced by a Gaussian distribution if the photon flux is >5 photons per pixel with <1% error, to conclude that the camera-related noise is the sum of two independent Gaussian-distributed random variables, with variance:

$$\sigma_N^2 = \sigma_R^2 + \sigma_G^2 \quad \text{(S9)}$$

where σ_G^2 represents the Gaussian variance of the photon shot noise. Under this approximation, the noise distribution has a constant power spectral density, while signals from the actual sample are contained within the Optical Transfer Function (OTF) of the system and are further modulated by the natural frequency f_0 of the rotating object. On the other hand, because the system is sampled at a frequency of f_s= fps (fps = 150 in our current analysis), a band of $0 < f < f_s/2$ can provide a complete reconstruction of the signal information by the Nyquist-Shannon sampling theorem. Temporal signal frequencies in the range of $f_0 \lesssim f \leq f_s/2$ can be therefore considered to be potentially parasitic. In practice, however, because a cut-off value close to the natural frequency of the system can result in over-filtering, we decided to keep frequencies at least approximately one order of magnitude above $2f_0$, which in the parametric study (Fig. 4F in the main manuscript) corresponded to f_c~ 5 Hz.

To improve signal quality prior to any digital filtering, the following noise reduction strategies are suggested:

- Increasing the quantum efficiency of the system – essentially, the efficiency by which impinging photons are transformed into photoelectrons. This can be achieved in a back illumination detector scheme with a back thinning (BT) process, typically present in more costly cameras (e.g. scientific grade CMOS sensors).

- Binning schemes can be used to combine the signal incoming from different contiguous pixels into one compound pixel, thereby increasing the number of impinging electrons per pixel and therefore the signal, with respect to a given noise level. A trade off would be that by combining pixels, resolution is reduced (e.g. by lowering the total number of pixels present in the final image).

- Longer exposure times can also be used to increase the signal level with respect to the noise levels of the system. This results in improved SNR but can critically impact the number of frames that can be collected per unit time. Reducing frame rate can ultimately result in a reduced number of optical slices, which are needed to perform the OPT-based 3D reconstruction.

- Enhancing the optics needed to acquire the images of rotating cells could also be used to improve SNR. In particular, an increase in the NA of the objective (currently NA = 1.3) would have a direct impact on the amount of collected photons by widening the light cone that enters the objective lens.

- Optimization of the imaging conditions within droplets could also lead to SNR improvements. Choosing cell media that provides low background fluorescence will reduce noise incoming from the sample. Furthermore, careful selection of the fluorophore excitation conditions of interest will have a direct impact on image quality. Excitation and emission filters can also be selected optimally for improved SNR, or laser excitation can be used instead to provide excitation photons closer to the specific wavelength needed for the fluorophore of interest.

Theory of shape representation via Spherical Harmonics (SPHARM)

The theory of SPHARM expansions for representing a 3D surface object $\mathbf{v}(\theta,\phi)$ has been previously described in detail (*4*, *5*). Briefly, each of the spatial coordinates (x,y,z) making up the vertices of the surface object can be expanded as a sum of spherical harmonic basis functions, $Y_l^m(\theta,\phi)$, where l represents the desired degree for the expansion and m the order of the spherical harmonic function:

$$\mathbf{v}(\theta,\phi) = \sum_{l=0}^{\infty}\sum_{m=-l}^{l}\mathbf{c}_l^m Y_l^m(\theta,\phi). \tag{S10}$$

where

$$\mathbf{v}(\theta,\phi) = \begin{pmatrix} x(\theta,\phi) \\ y(\theta,\phi) \\ z(\theta,\phi) \end{pmatrix}, \tag{S11}$$

$$\mathbf{c}_l^m = \begin{pmatrix} c_{xl}^m \\ c_{yl}^m \\ c_{zl}^m \end{pmatrix}. \tag{S12}$$

In these equations, the spherical harmonic functions are defined as:

$$Y_l^m(\theta,\phi) = \sqrt{\frac{2l+1}{4\pi}\frac{(l-m)!}{(l+m)!}}P_l^m(\cos\theta)\exp(im\phi) \tag{S13}$$

where P_l^m are the associated Legendre polynomials. Coefficients are calculated by computing the inner product of $\mathbf{v}(\theta,\phi)$ with the basis functions (*4*):

$$\begin{aligned}\mathbf{c}_l^m &= \langle\, \mathbf{v}(\theta,\phi)\,|Y_l^m(\theta,\phi)\rangle \\ &= \int_0^{\pi}\int_0^{2\pi}\mathbf{v}(\theta,\phi)\,Y_l^m(\theta,\phi)\mathrm{d}\phi\,\sin(\theta)\,\mathrm{d}\theta\end{aligned} \tag{S14}$$

Expansion of the 3D surface object into its Fourier basis functions is possible if and only if the object has genus zero. As an example, the x component of the vertices making up surface $\mathbf{v}(\theta,\phi)$ is given by (*6*):

$$x(\theta,\phi) = \sum_{l=0}^{\infty}\sum_{m=-l}^{l}c_{xl}^m Y_l^m(\theta,\phi). \tag{S15}$$

Existing Matlab implementations, such as the Spherical Harmonic Modeling and Analysis Toolkit (SPHARM-MAT Version 3.0) by Shen et al. (*5*), are available for calculation of the $\mathbf{c}_l^m$ coefficients of a given surface object $\mathbf{v}(\theta,\phi)$.

SPHARM-MAT data preparation. To analyze the fluorescence volumetric data obtained from ADOPT, we decided to build surface models from the different cell components under analysis. Upon collecting an OPT reconstructed object, a 3D matrix is generated, where each value represents the measured fluorescence intensity in a digital number (DN) format. These DN values represent translated photons from electrons with conversion factors dependent on the digital sensor (CMOS). To create the 3D surface models that would be analyzed by SPHARM, we normalized the intensity of said 3D matrices by their maximum intensity and applied a threshold to all values above a given isovalue. This operation essentially translates to indicating by a logical "1" all values within the fluorescence 3D matrices that are positive for the given fluorophore under analysis (Hoechst 33342 for all nuclei analyzed). This is a simple selection criterion, and while other more sophisticated methods may be used (e.g. through edge detection algorithms or

machine learning methods), the high SNR of the fluorophore lends itself to simple thresholding detection.

After obtaining the binary 3D matrix (`BW`) for each cell nucleus, a simple topological fix was performed on the data using Matlab's built-in function `imfill(BW, 'holes')` to guarantee simply connected objects prior to SPHARM analysis. This would guarantee all binary matrices had genus zero (i.e., no topological holes). Once topologically corrected, the binary matrices were transformed into two output vectors, one representing all vertices on the surface of the binary object and another representing their connectivity (faces) via function `isosurface(·)`. These vertex and face vectors, which essentially represent a triangular mesh of the voxels that were positive for the fluorophore of interest in 3D space, were then used as the input for the SPHARM algorithm.

SPHARM-MAT parameters. SPHARM utilizes a Fourier transform approach to represent a 3D surface with three spherical functions, converting them into three sets of Fourier coefficients within the frequency domain (*5*). Spherical Harmonic analysis was carried out using the stand-alone Matlab implementation of the spherical harmonic surface parametrization method by Brechbühler et al. (*4*), SPHARM-MAT developed by Shen et al. (*5*). First, SPHARM parametrization of the vertex and face vector of each cell nucleus was carried out using the Control of Area and Length Distortions (CALD) method. The following settings were selected: MeshGridSize: 50, MaxSPHARMDegree: 20, Tolerance: 2, Smoothing: 2, Iteration: 100, LocalIteration: 10, t_major: x, and SelectDiagonal: ShortDiag.

After parametrization, the output .mat files were fed into the "Expansion" algorithm using the following settings: Method: LSF, MaxSPHARMDegree: 20. This resulted in three sets of Fourier coefficients (one for each spatial cartesian coordinate). The total number of Fourier coefficients was given by $(L_{max}+1)^2$, where L_{max} is the MaxSPHARMDegree used in the reconstructions (here, $L_{max}=20$), for a total of 441 Fourier terms. To compare the coefficients of different nuclei (e.g. Fig. S3C), SPHARM alignment was carried out. First Order Ellipsoid (FOE) alignment was selected to establish surface correspondence.

Orientation-independent surface concavity measurement from SPHARM shapes. Because SPHARM coefficient determination requires surface matching and alignment to register 3D SPHARM model objects, we decided to follow an approach that would be independent of the orientation of the generated spherical harmonic expansions. With such a method, FOE alignment would not be necessary, while still providing a meaningful topological comparison between 3D organelle surface objects. To do this, we realized from observation of SPHARM models with different degrees of detail (i.e., L_{max}) that concave features arise at low frequency components, while high frequency components contain an almost 50/50 composition of concave and convex surfaces. This stems from the fact that the addition of harmonics up to high frequency components result in complex shaped surfaces with intricate detail that have at portions positive and negative curvatures. A property of SPHARM 3D object reconstructions is that they have smooth surfaces, and thus lend themselves to application of computational algorithms for calculation of their local Gaussian curvature. We applied existing Matlab algorithms (*7*, *8*) to calculate the areas having positive or negative curvature and proceeded to apply this method to each 3D surface nucleus analyzed for K562 cells and T cells. Therefore, to evaluate organelle concavity, we calculated the total portion of the organelle 3D surface having negative curvature:

$$\text{Area fraction with negative curvature in the } j\text{-th degree model} = \frac{A_{neg,j}}{A_{total,j}} = \frac{\sum_{i=1}^{N} A_{ij}\delta_i}{\sum_{i=1}^{N} A_{ij}} \quad \text{(S16)}$$

where A_{ij} represents the area of the i-th triangular face in the surface model of the j-th degree SPHARM model, N represents the total number of faces, and:

$$\delta_i = \begin{cases} 1\ , & \text{if } \kappa_{1ij}\kappa_{2ij} < 0 \\ 0, & \text{if } \kappa_{1ij}\kappa_{2ij} \geq 0 \end{cases} \ , \tag{S17}$$

with κ_{1ij}, κ_{2ij} being the principal curvature components of the i-th face in the j-th degree model. In this analysis, i spanned all the triangular faces (i=1 to $i = N$,with N=20,480 for all cases) in a surface model, and the degree of the SPHARM model was swept from j=1 to $j = L_{max}$=20. In Eq. (S16), the summation in the denominator corresponds to the total surface area of the j-th SPHARM degree surface model. Under the assumption that negative curvature surfaces represent the concavity, we evaluated Eqs. (S16) and (S17) for all K562 and T cell nuclei in this paper.

Orientation-independent surface concavity measurement validation. We validated that the concavity measurements obtained by SPHARM representation of cell nuclei were independent of the orientation of the raw 3D volumetric fluorescence (grayscale) data. To achieve this, random orientation states of the 3D volumetric fluorescence data were introduced by selecting 583 random orientation vectors and rotation angles (Fig. S4). This produced a data set of 583 randomly oriented 3D volumetric fluorescence matrices, which were transformed into their 3D surface voxel representation and then processed to produce the SPHARM shapes as described in the previous section. As seen from the mean and standard deviation values of the concavity measurement plots of Fig. S4C, harmonics above l = 6 result in less than 10% variation, as prescribed by the coefficient of variation: CV (%) = $\sigma/\mu \times$ 100%. For the maximum SPHARM degree (L_{max} = 20), variation was as low as 3.53%. This validation procedure thus provided an objective measurement of the orientation independence of concavity measurement through negative Gaussian area integration at each different harmonic level.

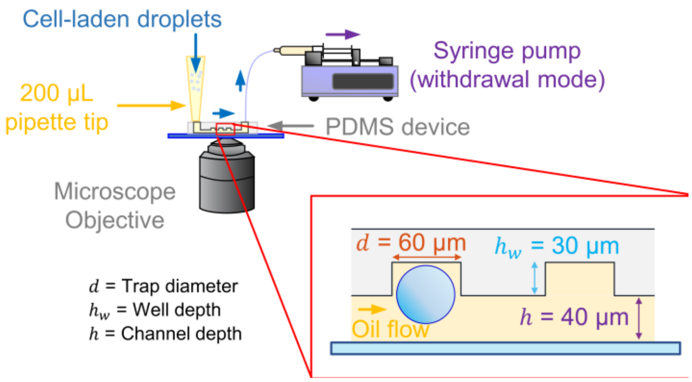

Fig. S1. Droplet trap device operation. Schematic showing the syringe pump operating in withdrawal mode to draw cell-laden droplets into the microfluidic trap array. A 200 µL pipette tip was used as reservoir to feed droplets into the device. A blow-up of the device side view depicts the relevant design dimensions used in this study.

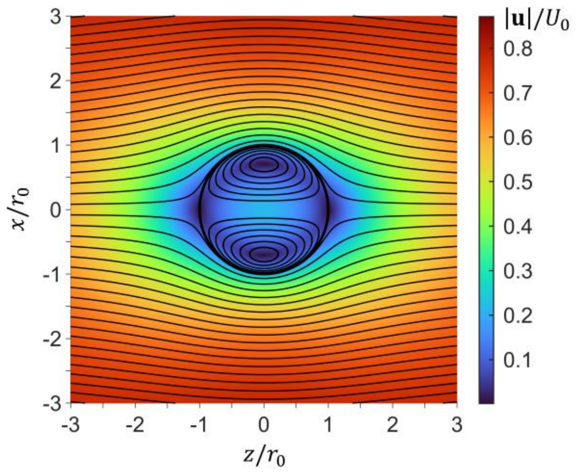

Fig. S2. Flow velocity field of a 3D spherical droplet. 2D colormap represents the magnitude of the flow velocity field, normalized by the outer flow velocity magnitude far from the droplet. The flow field has azimuthal symmetry. Far from the droplet, the flow field is equal to $-\boldsymbol{U_0}\hat{\mathbf{z}}$.

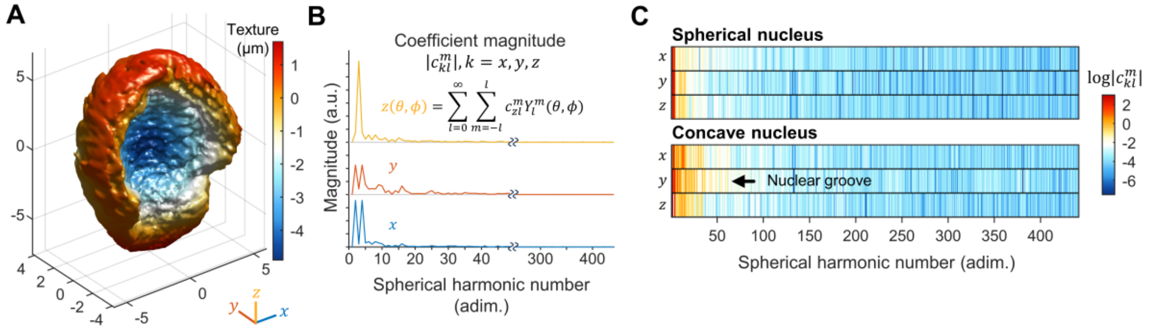

Fig. S3. Spherical harmonics representation of the cell nuclear textural information. (A) Texture map of the K562 cell nucleus (Fig. 2H in the main manuscript, included here for ease of visualization), displaying an enlarged nuclear groove structure. (B) Spherical harmonics parametric representation of the nucleus isosurface rendering in (A). (C) Magnitude of the spherical harmonic coefficients used in approximating spherically shaped and concave shaped K562 cell nuclei of Fig. 2K and L in the main manuscript. The spectral decomposition evinces the presence of the nuclear groove in the y-axis direction.

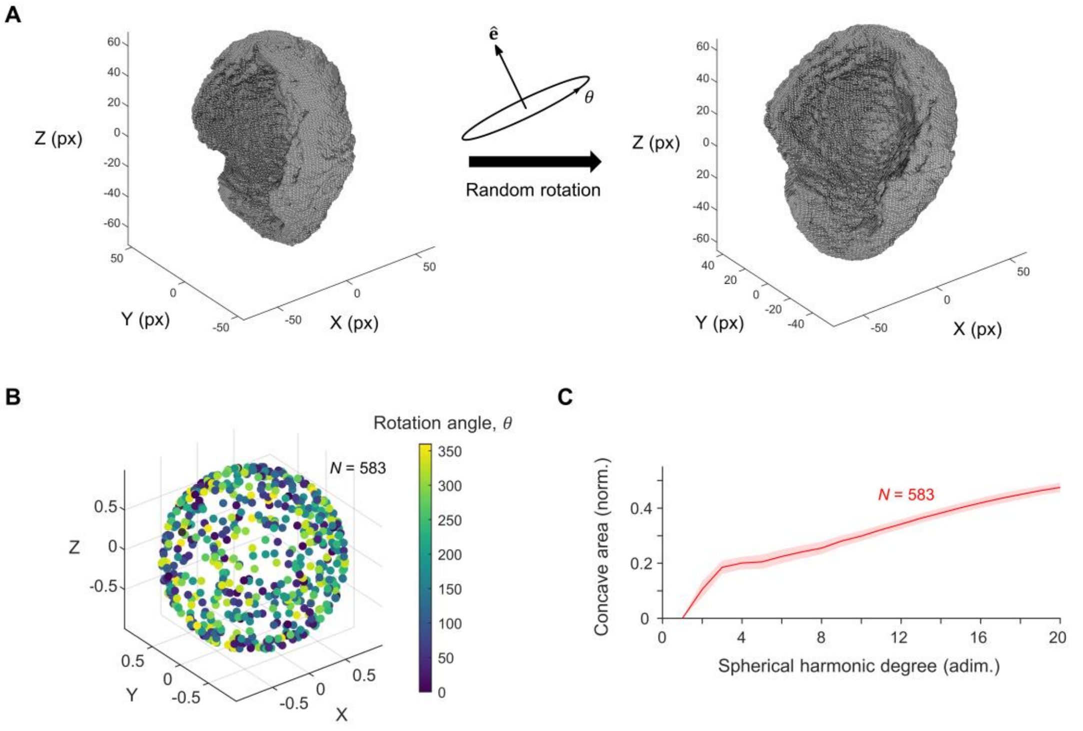

Fig. S4. Validation of orientation independence in cell organelle surface concavity measurements. (A) Schematic representation of a random rotation operation applied to a K562 cell nuclei (obtained from 3D fluorescence volumetric data). (B) Scatter plot showing the N=583 different random rotation operations here tested. Each point represents the coordinates of a random orientation vector, and the point color indicates the applied random rotation angle (0°-360°) about the axis described by the orientation vector. (C) Measured concavity area of each SPHARM representation of the shape in (A), with increasing number of harmonic degree expansion. For N=583 randomly oriented 3D fluorescence volumetric data sets (K562 cell nuclei), the concave nuclear area was measured at each level of harmonic degree representation and averaged, with the standard deviation recorded (upper and lower shaded bounds to the line plot in C).

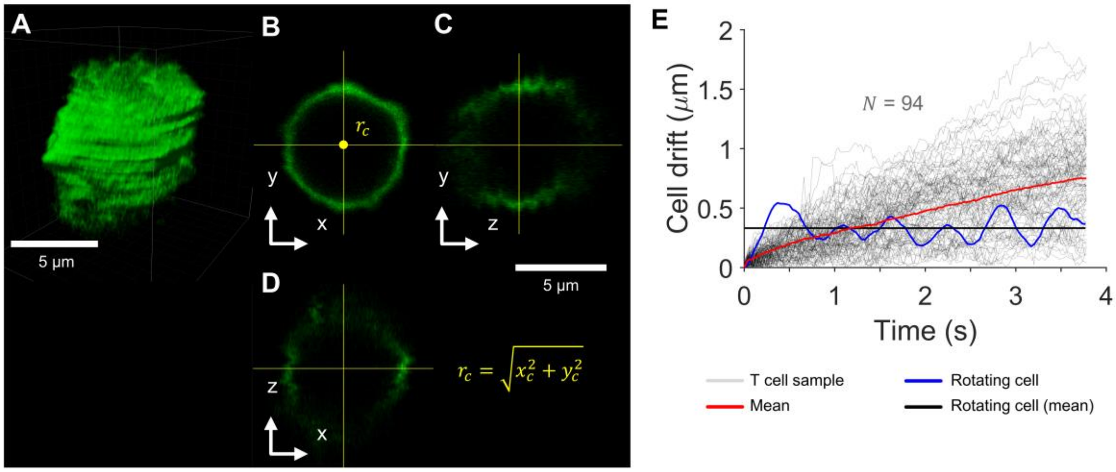

Fig. S5. Single-cell drift artifacts typically present in optical sectioning techniques. (A) Translation and rotation motion artifacts of unfixed T cells, as imaged by a CLSM system (fluorophore: anti-CD45-Alexa Fluor 488). (B) A single xy-plane slice of the T cell in (A), showing its central position for tracking. (C,D) Different plane cuts illustrating motion artifacts. (E) Cell drift as a function of time, traced for N = 94 T cells. The calculated drift (in the xy-plane) of the centroid of a rotating cell inside a droplet. Average values are highlighted to evince motion trends.

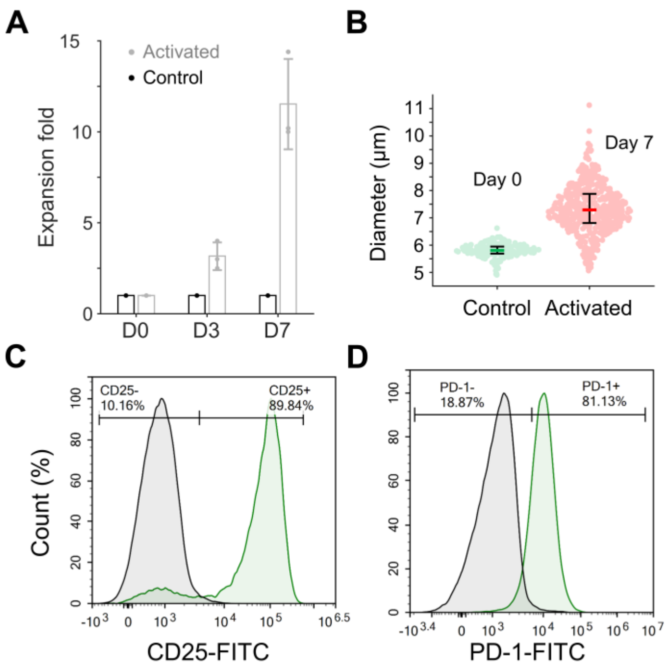

Fig. S6. Activated T cell phenotype. (A) Primary T cell expansion-fold as a function of elapsed days after activation, for N =3 healthy donors. (B) T cell size distribution change from day 0 to 7. (C,D) Example of T cell activation markers distributions. Flow cytometry analysis of the percent of (A) CD25+ and (B) PD-1+ pan T cells on day 3 post-activation, for blood donor 1. Error bars represent quartile bounds.

Movie S1 (separate file). Brightfield footage of a rotating K562 cell trapped inside droplet under 2 µL min^{-1} external oil phase flow rate. Scale bar = 20 µm.

Movie S2 (separate file). Brightfield footage of a rotating K562 cell trapped inside droplet under 10 µL min^{-1} external oil phase flow rate. Scale bar = 20 µm.

Movie S3 (separate file). A rotating K562 cell trapped inside droplet with its nucleus stained using Hoechst 33342. Video was captured at 150 fps using a DAPI filter in a conventional widefield epifluorescence microscope at 100x (NA=1.3). Scale bar = 4 µm.

Movie S4 (separate file). Reconstructed 3D surface model obtained via Optical Projection Tomography of raw footage in Movie S3. Surface was constructed via an isosurface rendering of the 3D volumetric fluorescence intensity profile. Scale bar = 4 µm.

SI References

1. J. S. Hadamard, Mouvement permanent lent d'une sphere liquid et visqueuse dans un liquide visqueux. *CR Hebd. Seances Acad. Sci. Paris* **152**, 1735–1738 (1911).
2. B. Mandracchia, W. Liu, X. Hua, P. Forghani, S. Lee, J. Hou, S. Nie, C. Xu, S. Jia, Optimal sparsity allows reliable system-aware restoration of fluorescence microscopy images. *Sci. Adv.* **9**, eadg9245 (2023).
3. B. Mandracchia, X. Hua, C. Guo, J. Son, T. Urner, S. Jia, Fast and accurate sCMOS noise correction for fluorescence microscopy. *Nat. Commun.* **11**, 94 (2020).
4. C. Brechbühler, G. Gerig, O. Kübler, Parametrization of Closed Surfaces for 3-D Shape Description. *Comput. Vis. Image Underst.* **61**, 154–170 (1995).
5. L. Shen, F. Makedon, Spherical mapping for processing of 3D closed surfaces. *Image Vis. Comput.* **24**, 743–761 (2006).
6. C.-J. Du, P. T. Hawkins, L. R. Stephens, T. Bretschneider, 3D time series analysis of cell shape using Laplacian approaches. *BMC Bioinformatics* **14**, 296 (2013).
7. S. Rusinkiewicz, "Estimating curvatures and their derivatives on triangle meshes" in *Proceedings. 2nd International Symposium on 3D Data Processing, Visualization and Transmission, 2004. 3DPVT 2004.* (2004), pp. 486–493.
8. Y. Ben Shabat, A. Fischer, Design of Porous Micro-Structures Using Curvature Analysis for Additive-Manufacturing. *Procedia CIRP* **36**, 279–284 (2015).
