# Supplementary figures and images for: Three-dimensional isotropic imaging of live suspension cells enabled by droplet microvortices
Source: Proc Natl Acad Sci U S A. 2024 Oct 22;121(44):e2408567121. doi: 10.1073/pnas.2408567121 (PMC11536124; doi:10.1073/pnas.2408567121)

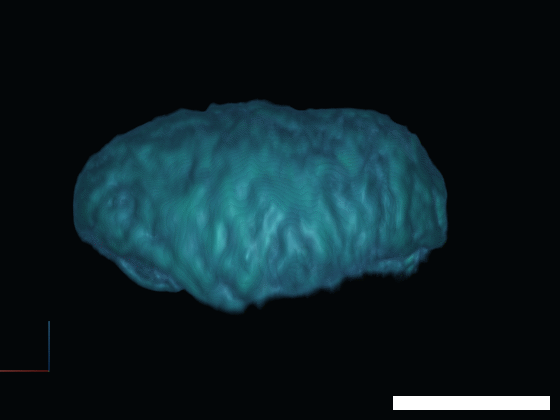

Supplement: Movie S4. — Reconstructed 3D surface model obtained via Optical Projection Tomography of raw footage in Movie S3. Surface was constructed via an isosurface rendering of the 3D volumetric fluorescence intensity profile. Scale bar = 4 μm. [file pnas.2408567121.sm04.gif]
